# Telegraphic Transport Processes and Their Fractional Generalization: A Review and Some Extensions

**DOI:** 10.3390/e23030364

**Published:** 2021-03-18

**Authors:** Jaume Masoliver

**Affiliations:** Department of Condensed Matter Physics and Complex Systems Institute (UBICS), University of Barcelona, 08007 Barcelona, Catalonia, Spain; jaume.masoliver@ub.edu

**Keywords:** telegrapher’s equations, fractional telegrapher’s equation, continuous time random walk, transport problems, 02.50.Ey, 05.40.Fb, 05.40.Jc, 05.60.Cd

## Abstract

We address the problem of telegraphic transport in several dimensions. We review the derivation of two and three dimensional telegrapher’s equations—as well as their fractional generalizations—from microscopic random walk models for transport (normal and anomalous). We also present new results on solutions of the higher dimensional fractional equations.

## 1. Introduction

In many physical situations particle transport through continuous media is described by transport equations which are typically derived from general physical principles as, for instance, the conservation of energy and momentum [1]. Classical cases are provided by the transport of neutron in a reactor or the photon transport in a highly scattering medium [2]. In their most general form transport equations (whose one of the first and most paradigmatic example is the Boltzmann equation) are nonlinear integro-differential equations often with an incompletely known scattering kernel [1,2]. It is therefore very difficult, not to say impossible, to attain exact analytical solutions of the problem and even obtaining numerical solutions is not an easy task. Moreover, numerical solutions might not reproduce, or even detect, important qualitative characteristics of the transport process [2].

These difficulties have traditionally lead to the search of simpler and easier way to handle approximations. One of the most universal approximation is modeling the transport process by diffusion processes. Such approximation greatly simplifies the description of the transport process because in the absence of any field driving the particle the usually complicated transport equation is reduced to the much simpler diffusion equation:

(1) $$\frac{\partial p}{\partial t}=D\nabla^{2}p,$$

here p(r,t) is the probability density function (PDF) of the diffusing particle to be at r at time *t* and *D* is the diffusion coefficient.

Diffusion processes have two major characteristics: (i) the mean square deviation grows linearly with time,

(2) $$\langle |\Delta r\left(t\right){|}^{2}\rangle =Dt,$$

where Δr(t)=r(t)−〈r(t)〉; (ii) the PDF is Gaussian. Indeed, the solution to Equation (1) assuming the particle is initially at the origin, p(r,0)=δ(r), is

(3) $$p\left(r,t\right)=\frac{1}{{\left(4\pi Dt\right)}^{3/2}}e^{-r^{2}/4Dt}.$$

Despite its simplicity and the wide range of applications in countless areas of physical sciences, the diffusion approximation has, however, several limitations. We will here point out two of them. First, diffusion processes present an infinite velocity of propagation. This can be easily seen from Equation (3) where it is shown that the solution p(r,t)>0 never vanishes for any finite time and distances r=|r|. There is, nonetheless, a nonzero probability (albeit small) of finding the diffusive particle, at any instant of time, arbitrarily far away from the initial position. In consequence diffusion models allow for arbitrary velocities, even larger than the speed of light in vacuum. This is contrary to the principles of relativity and certainly unsatisfactory from a conceptual point of view [3].

On the other hand, diffusion processes are also unable to account for ballistic motion and are rather useless in describing of early-time effects when ballistic motion may be important as well as near interfaces and in thin samples. This is certainly the case when modeling transport phenomena for which thermalization due to random collisions takes a finite time and the flux of ballistic particles might not be negligible, all of it resulting in anisotropic scattering along the forward direction. A particular but significant case is that of the photon migration through turbid media in which diffusion models are unable to account for ballistic photons and are inaccurate near boundaries [2,4,5,6]. A similar situation may arise in transport across membranes [7].

Telegraphic processes are a generalized form of diffusion processes in these two aspects. Thus (i) they allow for a finite velocity of propagation and (ii) they are nearly deterministic (i.e., ballistic) at short times while they are diffusive at long times when random collisions have been able to thermalize the motion. As a first approximation, the transport equation for telegraphic processes is the telegrapher’s equation (TE):

(4) $$\frac{\partial^{2}p}{\partial t^{2}}+\frac{1}{\tau }\frac{\partial p}{\partial t}=v^{2}\nabla^{2}p,$$

where τ>0 is a characteristic time, and v>0 is a characteristic speed. From a mathematical point of view, this is a hyperbolic equation which, as τ→∞ with *v* fixed, becomes the wave equation,

(5) $$\frac{\partial^{2}p}{\partial t^{2}}=v^{2}\nabla^{2}p,$$

while as τ→0 and v→∞ such that $v^{2}\tau \to D$ is finite it reduces to the diffusion Equation (1). The telegrapher’s equation thus possesses wave and diffusion features describing “diffusion with finite propagation velocity” but also “wave motion with damping” [8]. Moreover, the limits to diffusion and wave equations are also achieved as time progresses. We can thus easily see by scaling time with τ that initially as t→0 (i.e., t≪τ), TE approaches to the wave equation while asymptotically as t→∞ (t≫τ) moves toward the diffusion equation. As a consequence [2,8]

$${\langle |\Delta r\left(t\right)|}^{2}\rangle ∼t^{2}{,\;\;\left(t\to 0\right)\;\mathrm{and}\;\langle |\Delta r\left(t\right)|}^{2}\rangle ∼t,\;\;\left(t\to \infty \right),$$

which heightens the duality of the TE and shows the transition from ballistic motion to diffusive motion as time progresses.

The TE appeared in the nineteenth century in the works of Kelvin and Heaviside related to the analysis of transmission of electromagnetic waves in telegraphic wires. In this context, the three dimensional telegrapher’s equation can be derived by combining Maxwell’s equations for homogeneous media [2,8]. TE can also be phenomenologically derived from thermodynamics by using Cattaneo’s equation, a nonlocal generalization of Fick’s law accounting for non instantaneous diffusions [9,10,11], and also from random walk theory where the one-dimensional TE is the master equation of the persistent random walk [12,13,14,15].

From a mesoscopic point of view (somewhere between the microscopic view of random walk models and the macroscopic approach of thermodynamics) telegraphic processes are closely related to Brownian motion. As was studied some years ago in Ref. [16], the telegrapher’s equation, like the diffusion equation, can also be derived from the Chapman-Kolmogorov equation, which is the master equation for Markovian processes [17]. It is worth noticing that such a derivation is obtained by retaining quadratic terms in the time expansion of the Chapman-Kolmogorov equation which sets a characteristic time scale and a characteristic velocity. The Markovian character of the process is assured for times greater than the characteristic time while a possible non-Markovian character for smaller times s still an unsettled question [16].

In the context of transport theory, the three-dimensional TE is the so-called P_1_ approximation to the full transport equation for which the basic assumption is that the change in the direction of motion due to a single scattering event is small [1,2,18,19]. In a more recent approach [20] a three-dimensional TE model is obtained by a modification of the continuity equation for the probability current. The model is, however, limited to a discrete number of transport directions, which restricts possible applications. Other approaches suppose phenomenological generalizations, where a three dimensional TE is postulated for uniform isotropic media by assuming the same form as the one-dimensional TE, but with numerical corrections in the coefficients which guarantee correct ballistic (t→0) and diffusive (t→∞) behaviors in three dimensions [4,5,6]. The more fundamental and less phenomenological way of describing telegraphic processes is, however, based on random walk models since they try to reproduce the microscopic mechanism of transport.

Random walk models for describing telegraphic processes are modifications of the ordinary random walk because the latter, for long times and large distances (i.e., the so-called “fluid limit” [21]) leads to the diffusion equation but not to the telegrapher’s equation [2,8,22]. However, and contrary to one dimension where the TE is readily obtained from the persistent random walk on the line [2,12,14], in higher dimensions obtaining the TE from microscopic models encounters serious difficulties. The main reason lies in the difficulty of generalizing persistence in dimensions greater than one [23,24,25,26,27,28,29].

We have recently solved this problem by obtaining the three-dimensional TE [30] and the two-dimensional TE [31] from random walk models (as we had done previously for the one dimensional case [32]). These models consist of a continuous version of two and three dimensional random walks with a continuum of states [33]. I will here review and enlarge these works.

For more than two decades, the so-called “anomalous transport” and “anomalous diffusion” have been the object of intense research with countless applications in many areas of physics, chemistry and natural and socio-economic sciences. There is an immense literature on the subject with many complete reports. As a necessarily short sample we may cite from early reviews in [34,35,36,37,38,39] to more recent reports [40,41,42] among many others. It is also worthwhile mentioning a less technical but excellent introduction in [43]. The concept first appeared from the theory of random processes, specifically within continuous time random walks, a powerful technique developed by Montroll and Weiss more than 50 years ago [22,44,45] (see a recent and updated review in Ref. [46]) and it was first applied to diffusion of charge carriers in organic semiconductors by Scher and Montroll in the 1970’s [47,48].

Anomalous transport arises in motion through extremely disordered systems such as random media and fractal structures [49] and its most distinctive characteristic is that the mean square deviation follows the asymptotic law [35,36,50]

(6) $${\langle |\Delta r\left(t\right)|}^{2}\rangle ∼t^{\alpha },$$

(t→∞), where α>0 is any positive real number. When 0<α<1 the transport regime is subdiffusive, α=1 corresponds to diffusive transport while α>1 describes superdiffusion. Within the diffusive approximation and in the force-free case, the anomalous transport process is described by a fractional diffusion equation,

(7) $$\frac{\partial^{\alpha }p}{\partial t^{\alpha }}=D\nabla^{2\gamma }p$$

(0<α≤1,0<γ≤1), $\partial^{\alpha }/\partial t^{\alpha }$ is the fractional Caputo derivative and $\nabla^{2\gamma }$ is the Riesz–Feller fractional Laplacian (see Section 5.2 for a definition of these operators). In the case of particles diffusing under the influence of an external field of force, Equation (7) is replaced by a fractional Fokker–Plank equation [36,38].

The mathematical properties of the solutions to the fractional diffusion Equation (7) have been thoroughly studied and very clearly exposed by Mainardi, Gorenflo and collaborators [51,52,53]. One of these properties is the scaling relation [21,35,53]

(8) $$p\left(r,t\right)=t^{-\alpha /2\gamma }f\left(\frac{r}{t^{\alpha /2\gamma }}\right),$$

resulting in the mean square displacement [21]:

(9) $$\langle r^{2}\left(t\right)\rangle =Mt^{\alpha /\gamma }.$$

When γ=1 but α is not an integer we have the ‘time-fractional diffusion”; the case 0<α<1 corresponding to subdiffusion while α>1 to superdiffusion. When α=1 but γ is not integer, the fractional diffusion Equation (7) describes a Levy process, this case is always associated with superdiffusion and it is termed “space-fractional diffusion” [21,38].

As mentioned above, the original motivation for the fractional transport was devised from the continuous time random walk formalism [47,48]. As a result, the derivations of the fractional diffusion equation are mostly based on this formalism, although alternative approaches exist based on master equations or (fractional) Chapman–Kolmogorov expansions [36].

The fractional Equation (7) ignores changes in the dynamics of the diffusing particle as time increases. These changes account for ballistic motion and anisotropic scattering (among others) that are relevant in a number of experimental settings [54]. The TE explains some of these characteristics of transport which imply the transition form ballistic to diffusive motion asymptotically in time.

In a recent work [32] we have presented a derivation of the fractional telegrapher’s equation (FTE) in one dimension based on a fractional generalization of the persistent random walk on the line. The continuous multistate model mentioned above allows for a fractional treatment which finally leads to fractional TEs in higher dimensions [30,31].

In this paper we review all these questions and present some new results. The paper is organized as follows. In Section 2 we present the continuous multistate random walk in three dimensions, which in homogeneous and isotropic cases, allows us to derive the three-dimensional telegrapher’s equation (Section 3). In Section 4 we adapt the model to two dimensions and derive the corresponding telegrapher’s equation. The rest of the paper is devoted to the fractional generalization of these matters. In Section 5 we set the general model for fractional telegraphic transport and obtain the space-time fractional telegrapher’s equation in two and three dimensions along with the exact expression for the characteristic function. In Section 6 we study in detail the time-fractional telegrapher’s equation, analyze its solution for any dimensionality, and obtain asymptotic results for the probability distribution and the moments of the distance travelled. Concluding remarks are presented in Section 7.

## 2. Continuous Multistate Random Walk in Three Dimensions

We review the microscopic model introduced in Ref. [30] for the transport of particles in continuous media. The model is based on a generalization of multistate random walks and assumes a continuum in the number of states [33]. In the traditional formulation of multistate random walks (see [15] for a recent review on multistate walks on the line) the walker can be in a discrete (but not necessarily finite) number of internal states. The transition between states is determined by a transition matrix with random Markovian elements. In order to model particle transport we will generalize the multistate random walk in two key features: (i) we assume that the walker (i.e., the particle) moves in three dimensions, and (ii) the model has internal states defined on a continuous set of values.

### 2.1. General Setting

Suppose a particle moving in the three dimensional space along a straight line determined by the bidimensional quantity Ω=(θ,φ), where θ is the polar angle and φ is the azimuthal angle. The particular direction along which the particle is moving constitutes the “internal state” and since all possible direction form a continuous and denumerable set, the motion of the particle is thus described by a *continuous multistate random walk*.

At random instants of time the particle shifts direction and, hence, the duration of the motion along a given direction Ω (which is called a *sojourn*) is a random variable determined by a PDF denoted by ψ(t|Ω). The cumulative distribution

(10) $$\Psi \left(t|\Omega \right)=\int_{t}^{\infty }\psi \left(t^{\prime }|\Omega \right)dt^{\prime },$$

gives the probability that the duration of a given sojourn is greater than *t*.

Let us denote by h(r,t|Ω) the joint PDF for the displacement in a single sojourn along direction Ω to be equal to r and the sojourn duration to equal *t*. Let us also define H(r,t|Ω) as the probability density for the displacement to be r when the duration is greater than *t*. Note that the duration PDF ψ(t|Ω) is the time marginal density of h(r,t|Ω),

(11) $$\int_{R^{3}}h\left(r,t|\Omega \right)d^{3}r=\psi \left(t|\Omega \right),$$

while Ψ(t|Ω) is the marginal probability arising from H(r,t|Ω),

(12) $$\int_{R^{3}}H\left(r,t|\Omega \right)d^{3}r=\Psi \left(t|\Omega \right).$$

At the end of a given sojourn, the particle moving along direction $\Omega^{\prime }$ switches to direction Ω. We denote by $\beta (\Omega |\Omega^{\prime })$ the PDF of this transition $\Omega^{\prime }\to \Omega$ (note that $\beta (\Omega |\Omega^{\prime })$ is the “scattering kernel” of the transport problem). In other words, the probability that a single scattering changes the direction of the particle from $\Omega^{\prime }$ to a direction falling somewhere inside the angular region (Ω,Ω+dΩ) is given by

(13) $$\mathrm{Prob}\left\{\Omega^{\prime }\to \left(\Omega ,\Omega +d\Omega \right)\right\}=\beta \left(\Omega |\Omega^{\prime }\right)d^{2}\Omega ,$$

where dΩ=(dθ,dφ) and

(14) $$d^{2}\Omega =\sin\theta d\theta d\varphi$$

is the surface element on the sphere of unit radius.

Note that in this model there is a nonvanishing probability of traveling along the same direction and in those cases where this probability is greater than 1/2 the particle tends to persist in moving along the same direction. In this way the model can be seen as a higher dimensional generalization of the persistent random walk on the line [22].

Let us denote by p(r,Ω,t) the joint PDF for the walker to be at r at time *t* while moving in direction Ω. Our final objective is, however, to know the density p(r,t) for the random walker to be at r at time *t* regardless the direction. The latter is the marginal density of the former,

(15) $$p\left(r,t\right)=\int p\left(r,\Omega ,t\right)d^{2}\Omega .$$

In order to evaluate p(r,Ω,t) we define the auxiliary density ρ(r,Ω,t) as

$$\begin{array}{ccc} \rho \left(r,\Omega ,t\right)d^{3}rdt & = & \mathrm{Prob}\{a\;\mathrm{sojourn}\;\mathrm{in}\;\mathrm{direction}\;\Omega \;\mathrm{ends}\;\\ & & \;\mathrm{in}\;\mathrm{the}\;\mathrm{region}\;(r,r+dr)\;\mathrm{at}\;(t,t+dt)\}. \end{array}$$

This joint density describes the state of the process at the scattering points where the direction of the particle changes. Thus, if a scattering event happens at time t, it must either be the first one (assuming the initial one occurred at t=0) or else an earlier change of direction $\Omega^{\prime }\to \Omega$ [governed by $\beta (\Omega |\Omega^{\prime })$] happened at any earlier time $t^{\prime }<t$ with the random walker at some position $r^{\prime }$. It is not difficult to convince oneself that this renewal argument leads to the following integral equation for the auxiliary density:

(16) $$\begin{array}{ccc} \rho (r,\Omega ,t) & = & \beta (\Omega )h(r,t|\Omega )\\ & & +\int \beta \left(\Omega |\Omega^{\prime }\right)d^{2}\Omega^{\prime }\int_{0}^{t}dt^{\prime }\int_{R^{3}}h\left(r-r^{\prime },t-t^{\prime }|\Omega \right)\rho \left(r^{\prime },\Omega^{\prime },t^{\prime }\right)d^{3}r^{\prime }, \end{array}$$

where β(Ω) is the probability that the process starts moving in direction Ω.

In terms of the auxiliary density ρ(r,Ω,t), the PDF p(r,Ω,t) for the walker to be at r at time *t* while moving in direction Ω is

(17) $$\begin{array}{ccc} p(r,\Omega ,t) & = & \beta (\Omega )H(r,\Omega ,t)\\ & & +\int \beta \left(\Omega |\Omega^{\prime }\right)d^{2}\Omega^{\prime }\int_{0}^{t}dt^{\prime }\int_{R^{3}}H\left(r-r^{\prime },t-t^{\prime }|\Omega \right)\rho \left(r^{\prime },\Omega^{\prime },t^{\prime }\right)d^{3}r^{\prime }. \end{array}$$

The reasoning behind this equation is similar to the one given for obtaining Equation (16). Indeed, the displacement of the walker is either within the first sojourn, this given by βH, or else an earlier change of direction occurred at time $t^{\prime }<t$ while the walker was at position $r^{\prime }$ and the time interval to the next scattering exceeded $t-t^{\prime }$.

We thus see that in the most general case the solution to the problem, that is to say, obtaining the PDF p(r,t) (cf. Equation (15)) is given by first solving the integral Equation (16) for the auxiliary function ρ and afterwards substituting this solution into Equation (17) and the result into Equation (15). In the most general case, for arbitrary forms of $\beta (\Omega |\Omega^{\prime })$, h(r,t,|Ω) and H(r,t|Ω), obtaining analytical expressions is out of reach, and one has to resort to numerical work.

### 2.2. Independent Scattering

In order to proceed further we assume that after each scattering the direction is randomized independently of the previous direction of the particle leading to the scattering kernel:

(18) $$\beta \left(\Omega |\Omega^{\prime }\right)=\beta \left(\Omega \right).$$

The scattering process is thus an independent random process in the change of direction. In the context of fluctuations in laser fields this model corresponds to the so-called Burshtein model [55,56].

When the scattering kernel has the form given by (18), Equations (16) and (17) reduce to

(19) $$\rho \left(r,\Omega ,t\right)=\beta \left(\Omega \right)\left[h\left(r,t|\Omega \right)+\int_{0}^{t}dt^{\prime }\int_{R^{3}}h\left(r-r^{\prime },t-t^{\prime }|\Omega \right)d^{3}r^{\prime }\int \rho \left(r^{\prime },t^{\prime }|\Omega^{\prime }\right)d^{2}\Omega^{\prime }\right],$$

and

(20) $$\begin{array}{c} p\left(r,\Omega ,t\right)=\beta \left(\Omega \right)\left[H\left(r,t|\Omega \right)+\int_{0}^{t}dt^{\prime }\int_{R^{3}}H\left(r-r^{\prime },t-t^{\prime }|\Omega \right)d^{3}r^{\prime }\int \rho \left(r^{\prime },t^{\prime }|\Omega^{\prime }\right)d^{2}\Omega^{\prime }\right]. \end{array}$$

Integrating Equations (19) and (20) with respect to all possible directions Ω, defining the direction-free densities (cf. Equation (15))

(21) $$p\left(r,t\right)=\int p\left(r,\Omega ,t\right)d^{2}\Omega ,\;\;\rho \left(r,t\right)=\int \rho \left(r,\Omega ,t\right)d^{2}\Omega ,$$

and the averages

(22) $$h\left(r,t\right)=\int \beta \left(\Omega \right)h\left(r,\Omega ,t\right)d^{2}\Omega ,\;\;H\left(r,t\right)=\int \beta \left(\Omega \right)H\left(r,\Omega ,t\right)d^{2}\Omega ,$$

we get a simpler integral equation for ρ(r,t):

(23) $$\rho \left(r,t\right)=h\left(r,t\right)+\int_{0}^{t}dt^{\prime }\int_{R^{3}}h\left(r-r^{\prime },t-t^{\prime }\right)\rho \left(r^{\prime },t^{\prime }\right)d^{2}r^{\prime },$$

and the PDF p(r,t) will be given by

(24) $$p\left(r,t\right)=H\left(r,t\right)+\int_{0}^{t}dt^{\prime }\int_{R^{3}}H\left(r-r^{\prime },t-t^{\prime }\right)\rho \left(r^{\prime },t^{\prime }\right)d^{2}r^{\prime }.$$

The problem can now be solved in Fourier–Laplace space. Thus, defining the joint Fourier and Laplace transform,

$$\hat{\tilde{\rho }}\left(\omega ,s\right)=\int_{0}^{\infty }e^{-st}dt\int_{R^{3}}e^{i\omega \cdot r}\rho \left(r,t\right)d^{3}r,$$

the integral Equation (23) turns into a simple algebraic equation for $\hat{\tilde{\rho }}$ whose solution can be readily obtained and reads

(25) $$\hat{\tilde{\rho }}\left(\omega ,s\right)=\frac{\hat{\tilde{h}}\left(\omega ,s\right)}{1-\hat{\tilde{h}}\left(\omega ,s\right)}.$$

On the other hand, by transforming Equation (24) we get

$$\hat{\tilde{p}}\left(\omega ,s\right)=\hat{\tilde{H}}\left(\omega ,s\right)\left[1+\hat{\tilde{\rho }}\left(\omega ,s\right)\right],$$

which after substituting for (25) yields

(26) $$\hat{\tilde{p}}\left(\omega ,s\right)=\frac{\hat{\tilde{H}}\left(\omega ,s\right)}{1-\hat{\tilde{h}}\left(\omega ,s\right)},$$

The form of Equation (26) can be considered a generalization of the Montroll–Weiss equation [44,45] for higher dimensional continuous time random walks with independent directions.

### 2.3. The Isotropic and Uniform Random Walk

Equation (26) furnishes the formal solution to the transport problem for independent scattering in Fourier–Laplace space and it is valid for any form of the conditional densities h(r,t|Ω) and H(r,t|Ω) which describe the displacement inside a given sojourn in direction Ω. In other words, Equation (26) applies to any kind of motion inside a given sojourn and to any distribution of sojourn times. In order to proceed further and solve the problem in a specific way by obtaining the explicit expression for p(r,t) in real time and space, we first assume that the particle moves in an isotropic medium so that the pausing time density and its cumulative probability are independent of the direction,

ψ(t|Ω)=ψ(t),Ψ(t|Ω)=Ψ(t).

We next assume that inside any sojourn the motion is uniform with a constant speed *c* so that after each sojourn the velocity of the particle takes a different direction but with the same modulus and, hence, the kinetic energy is conserved. Despite its simplicity, the model describes the motion of noninteracting particles—such as, for instance, photons—undergoing elastic dispersion with fixed centers randomly distributed. The assumption of uniform motion leads to conclude that the conditional densities for the displacement inside a given sojourn have the form

(27) h(r,t|Ω)=δ(r−ctu)ψ(t),H(r,t|Ω)=δ(r−ctu)Ψ(t),

where u is the unit vector pointing in direction Ω=(θ,φ), that is

(28) u=(sinθcosφ,sinθsinφ,cosθ).

The Fourier transforms of these densities read

(29) $$\tilde{h}\left(\omega ,t|\Omega \right)=\psi \left(t\right)e^{i(\omega \cdot u)ct},\;\;\tilde{H}\left(\omega ,t|\Omega \right)=\Psi \left(t\right)e^{i(\omega \cdot u)ct}.$$

In addition to the assumption that after each collision the new direction of the particle is randomized independently of the previous direction (cf. Equation (18)), we also suppose *complete isotropy* in the sense that all outgoing directions are equally likely. For the three dimensional motion this implies

(30) $$\beta \left(\Omega |\Omega^{\prime }\right)=\beta \left(\Omega \right)=\frac{1}{4\pi }.$$

The characteristic function of the displacement inside any sojourn independent of the direction is given by the average

$$\tilde{h}\left(\omega ,t\right)=\int \tilde{h}\left(\omega ,t|\Omega \right)\beta \left(\Omega \right)d^{2}\Omega .$$

In the isotropic case and for uniform motion (cf. Equations (14), (29) and (30)) we have

$$\tilde{h}\left(\omega ,t\right)=\frac{1}{4\pi }\psi \left(t\right)\int e^{i(\omega \cdot u)ct}d^{2}\Omega .$$

That is,

$$\tilde{h}\left(\omega ,t\right)=\frac{1}{2}\psi \left(t\right)\int_{0}^{\pi }e^{i|\omega |ct\cos\theta }\sin\theta d\theta ,$$

which after integrating yields

(31) $$\tilde{h}\left(\omega ,t\right)=\psi \left(t\right)\frac{\sin|\omega |ct}{|\omega |ct}.$$

Analogously

(32) $$\tilde{H}\left(\omega ,t\right)=\Psi \left(t\right)\frac{\sin|\omega |ct}{|\omega |ct}.$$

In order to obtain the Fourier–Laplace transform of the PDF of the particle to be at position r at time *t* by means of Equation (26), we have to specify the form of the pausing time density ψ(t). One of the most natural and universal assumptions consists in taking the random instants of time at which the scattering process occurs to be a Poissonian set of events which implies that time intervals inside any sojourn are exponentially distributed [57]. Thus

$$\psi \left(t\right)=\lambda e^{-\lambda t}\;\Rightarrow \;\Psi \left(t\right)=e^{-\lambda t},$$

where $\lambda^{-1}$ is the average time interval between two consecutive scattering events (i.e., the mean sojourn duration). We have

$$\tilde{h}\left(\omega ,t\right)=\lambda e^{-\lambda t}\frac{\sin|\omega |ct}{|\omega |ct},\;\tilde{H}\left(\omega ,t\right)=\frac{1}{\lambda }\tilde{h}\left(\omega ,t\right).$$

We next take the Laplace transform of these expressions. Recalling that [58]

$$L\left\{\frac{\sin|\omega |ct}{t}\right\}=\arctan\left(\frac{|\omega |c}{s}\right),$$

and the property $L\left\{e^{-\lambda t}f\left(t\right)\right\}=\hat{f}\left(\lambda +s\right)$, we get

(33) $$\hat{\tilde{h}}\left(\omega ,s\right)=\frac{\lambda }{|\omega |c}\arctan\left(\frac{|\omega |c}{\lambda +s}\right)$$

and

(34) $$\hat{\tilde{H}}\left(\omega ,s\right)=\frac{1}{|\omega |c}\arctan\left(\frac{|\omega |c}{\lambda +s}\right).$$

Substituting Equations (33) and (34) into Equation (26) we finally get

(35) $$\hat{\tilde{p}}\left(\omega ,s\right)=\frac{\arctan[|\omega |c/(\lambda +s)]}{\left|\omega |c-\lambda \arctan[|\omega |c/(\lambda +s)\right]},$$

which constitutes the exact solution of the homogeneous and isotropic model and the starting point for deriving the three dimensional telegrapher’s equation as we will see next. It is worth mentioning that a similar expression was obtained some years ago by Claes and Van den Broeck [59] in the context of modeling the end-to-end distance of polymer chains, although they used a different approach.

## 3. Telegrapher’s Equation

The homogeneous and isotropic random walk reviewed is a microscopic model of particle transport. We can construct the TE from this model by coarse graining the dynamics to the fluid limit approximation.

### 3.1. Fluid Limit Approximation

The fluid limit approximation consists in rewriting the model for large times and distances [21,51]. Because of Tauberian theorems [60,61], large times and distances, t→∞ and |r|→∞, correspond to small Laplace and Fourier variables, s→0 and |ω|→0. Note that to achieve such a limit, i.e., to get an approximate expression for the transformed PDF $\hat{\tilde{p}}\left(\omega ,s\right)$ for small values of *s* and |ω|, we have two different and equivalent ways of proceeding. We can thus proceed either through the direct expansion of $\hat{\tilde{p}}$ given by Equation (35) or else through the expansions of $\hat{\tilde{h}}$ and $\hat{\tilde{H}}$ (cf. Equations (33) and (34)) as s→0 and |ω|→0 and their subsequent substitution in Equation (26). Obviously both procedures yield the same result but, albeit longer, we follow the second approach since it turns to be instrumental for the fractional generalization of the random walk.

We thus start off with Equation (33) and first perform the long-distance limit (|ω|→0) and postpone for a moment the long-time limit (s→0). As |ω|→0 we have the following expansion

(36) $$\begin{array}{ccc} \arctan\left(\frac{|\omega |c}{\lambda +s}\right) & = & \frac{|\omega |c}{\lambda +s}-\frac{1}{3}{\left(\frac{|\omega |c}{\lambda +s}\right)}^{3}+{O(|\omega |}^{5})\\ & = & \frac{|\omega |c}{{\left(\lambda +s\right)}^{3}}\left[{\left(\lambda +s\right)}^{2}-\frac{1}{3}{\left(|\omega |c\right)}^{2}+{O(|\omega |}^{4})\right]. \end{array}$$

From Equations (33), (34) and (36) we write

(37) $$\hat{\tilde{h}}\left(\omega ,s\right)=\frac{\lambda }{{\left(\lambda +s\right)}^{3}}\left[{\left(\lambda +s\right)}^{2}-\frac{1}{3}{\left|\omega \right|}^{2}c^{2}+{O(|\omega |}^{4})\right],$$

and

(38) $$\hat{\tilde{H}}\left(\omega ,s\right)=\frac{1}{{\left(\lambda +s\right)}^{3}}\left[{\left(\lambda +s\right)}^{2}-\frac{1}{3}{\left|\omega \right|}^{2}c^{2}+{O(|\omega |}^{4})\right].$$

Hence

$$\begin{array}{ccc} 1-\hat{\tilde{h}}\left(\omega ,s\right) & = & 1-\frac{\lambda }{{\left(\lambda +s\right)}^{3}}\left[{\left(\lambda +s\right)}^{2}-\frac{1}{3}{\left|\omega \right|}^{2}c^{2}+{O(|\omega |}^{4})\right]\\ & = & \frac{1}{{\left(\lambda +s\right)}^{3}}\left[{\left(\lambda +s\right)}^{3}-\lambda {\left(\lambda +s\right)}^{2}+\frac{\lambda }{3}{\left|\omega \right|}^{2}c^{2}+{O(|\omega |}^{4})\right]\\ & = & \frac{1}{{\left(\lambda +s\right)}^{3}}\left[s{\left(\lambda +s\right)}^{2}+\frac{\lambda }{3}{\left|\omega \right|}^{2}c^{2}+{O(|\omega |}^{4})\right], \end{array}$$

and as s→0, we may write

(39) $$1-\hat{\tilde{h}}\left(\omega ,s\right)=\frac{1}{{\left(\lambda +s\right)}^{3}}\left[s\left(\lambda^{2}+2\lambda s\right)+\frac{\lambda }{3}{\left|\omega \right|}^{2}c^{2}+O(s^{3}{,|\omega |}^{4})\right].$$

Substituting Equations (38) and (39) into Equation (26) yields

(40) $$\hat{\tilde{p}}\left(\omega ,s\right)=\frac{{\left(\lambda +s\right)}^{2}-{\left(c|\omega |\right)}^{2}/3+{O(|\omega |}^{4})}{s\left(\lambda^{2}+2\lambda s\right)+\lambda {\left(c|\omega |\right)}^{2}/3+O(s^{3}{,|\omega |}^{4})}.$$

In order to ensure the stability of Equation (40) under Fourier-Laplace inversion [and, hence, for the existence of a valid approximation for p(r,t)], it is necessary that the powers of *s* and |ω| which appear in the numerator of Equation (40) be less than the corresponding powers of the denominator [60]. We, therefore, write

$$\hat{\tilde{p}}\left(\omega ,s\right)=\frac{\lambda^{2}+2\lambda s+O(s^{2}{,|\omega |}^{2})}{s\left(\lambda^{2}+2\lambda s\right)+\lambda {\left(c|\omega |\right)}^{2}/3+O(s^{3}{,|\omega |}^{4})},$$

that is,

$$\hat{\tilde{p}}\left(\omega ,s\right)=\frac{\lambda /2+s+O(s^{2},|\omega {|}^{2})}{s\left(\lambda /2+s\right)+c^{2}{\left|\omega \right|}^{2}/6+O(s^{3}{,|\omega |}^{4})},$$

and take as a fluid limit approximation of the PDF the expression

(41) $$\hat{\tilde{p}}\left(\omega ,s\right)=\frac{s+\lambda /2}{s\left(s+\lambda /2\right)+c^{2}{\left|\omega \right|}^{2}/6}.$$

### 3.2. The Three-Dimensional Telegrapher’s Equation

Equation (41) is the starting point for deriving the two-dimensional TE. We next obtain the associated partial differential equation for p(r,t) whose solution, in Fourier–Laplace space and with appropriate initial conditions, is precisely given by Equation (41). To this end we multiply both sides of Equation (41) by the denominator and rewrite the result as

$$s^{2}\hat{\tilde{p}}\left(\omega ,s\right)-s+\frac{\lambda }{2}\left[s\hat{\tilde{p}}\left(\omega ,s\right)-1\right]=-\frac{c^{2}}{6}{\left|\omega \right|}^{2}\hat{\tilde{p}}\left(\omega ,s\right).$$

We now proceed to Fourier inversion. Taking into account

$$F^{-1}{\left\{|\omega \right|}^{2}\hat{\tilde{p}}\left(\omega ,s\right)\}=-\nabla^{2}\hat{p}\left(r,s\right),\;\;F^{-1}\left\{1\right\}=\delta \left(r\right),$$

the Fourier inversion yields

$$s^{2}\hat{p}\left(r,s\right)-s\delta \left(r\right)+\frac{\lambda }{2}\left[s\hat{p}\left(r,s\right)-\delta \left(r\right)\right]=\frac{c^{2}}{6}\nabla^{2}\hat{p}\left(r,s\right).$$

Let us next address Laplace inversion. With the standard initial conditions [62]

(42) $$p\left(r,0\right)=\delta \left(r\right),\;{\left.\frac{\partial p(r,t)}{\partial t}\right|}_{t=0}=0,$$

and the Laplace inversion formulas [58]

$$L^{-1}\left\{s^{2}\hat{p}\left(r,s\right)-s\delta \left(r\right)\right\}=\frac{\partial^{2}p\left(r,t\right)}{\partial t^{2}},$$

$$L^{-1}\left\{s\hat{p}\left(r,s\right)-\delta \left(r\right)\right\}=\frac{\partial p(r,t)}{\partial t},$$

we find that p(r,t) satisfies the three-dimensional TE

(43) $$\frac{\partial^{2}p}{\partial t^{2}}+\frac{1}{\tau }\frac{\partial p}{\partial t}=v^{2}\nabla^{2}p,$$

with

(44) $$\tau =1/\left(2\lambda \right)\;\mathrm{and}\;v=c/\sqrt{6},$$

as characteristic time and velocity respectively.

TE (43) enjoys both wave and diffusion characteristics. This duality becomes even more apparent as time progresses. Thus, as t→0 Equation (43) reduces to the wave equation while as t→∞ it goes to the diffusion equation. Indeed, scaling time with τ one can easily see that [15,30]

$$\frac{\partial^{2}p}{\partial t^{2}}≃v^{2}\nabla^{2}p\;\left(t\to 0\right),\;\;\frac{\partial p}{\partial t}≃D\nabla^{2}p\;\left(t\to \infty \right)$$

($D=v^{2}\tau$) which leads to

$${\langle |r\left(t\right)|}^{2}\rangle ∼t^{2}{\;\left(t\to 0\right),\;\langle |r\left(t\right)|}^{2}\rangle ∼t\;\left(t\to \infty \right),$$

showing the transition from ballistic motion to diffusive motion as time increases.

## 4. The Two Dimensional Case

Up to this point we have developed the telegraphic approximation to transport in three dimensions. We will now briefly report on how to treat the problem in lower dimensions.

In one dimension the standard derivation of the TE is based on the persistent random walk on the line [14]. In this model there is only one possible direction and the walker has two possible states since it can move either to the left or to the right with equal probability which is the isotropic case for the one dimensional motion. We do not present the details for the one-dimensional case here, but instead refer the interested reader to Ref. [15] for a recent and rather complete report. Note that the TE obtained is

(45) $$\frac{\partial^{2}p}{\partial t^{2}}+\frac{1}{\tau }\frac{\partial p}{\partial t}=v^{2}\frac{\partial^{2}p}{\partial x^{2}},ƒ$$

where in this case v=c coincides with the velocity of the moving particle and, as in three dimensions, $\tau ={\left(2\lambda \right)}^{-1}$ (recall that $\lambda^{-1}$ is the mean sojourn time when switching times are Poissonian).

### 4.1. General Model

The two-dimensional case has been recently developed in Ref. [31]. This microscopic model for transport in planar media has many similarities (but some particular differences) with the three dimensional model presented above. Let us note that now the direction of the particle is not given by the solid angle Ω=(θ,φ) but by the planar angle φ. Therefore, the equations for the continuous multistate random walk in two dimensions will be the same as those in three dimensions (cf. Section 2) with the replacements

(46) $$\Omega ⟶\varphi ,\;\;\int d^{2}\Omega ⟶\int_{0}^{2\pi }d\varphi ,\;\;\int_{R^{3}}d^{3}r⟶\int_{R^{2}}d^{2}r.$$

Thus, in the most general case the bidimensional model will be described by Equations (16) and (17) with these replacements in which the change of direction is governed by the transition density

$$\beta \left(\varphi |\varphi^{\prime }\right)d\varphi =\mathrm{Prob}\left\{\varphi^{\prime }\to \varphi +d\varphi \right\},$$

with similar definitions as those of the three dimensional walk for the densities ψ(t|φ), Ψ(t|φ), h(r,t|φ) and H(r,t|φ) (cf. Section 2.1).

For the case of independent scattering (Section 2.2)

$$\beta \left(\varphi |\varphi^{\prime }\right)=\beta \left(\varphi \right).$$

As in three dimensions, we can now also define direction-free densities by means of Equations (21) and (22) through replacements (46). Finally, the Fourier–Laplace transform of the PDF,

$$\hat{\tilde{p}}\left(\omega ,s\right)=\int_{0}^{\infty }e^{-st}dt\int_{R^{2}}e^{i\omega \cdot r}p\left(r,t\right)d^{2}r,$$

is explicitly given by the generalization of Montroll–Weiss Equation (26),

(47) $$\hat{\tilde{p}}\left(\omega ,s\right)=\frac{\hat{\tilde{H}}\left(\omega ,s\right)}{1-\hat{\tilde{h}}\left(\omega ,s\right)},$$

where $\hat{\tilde{h}}\left(\omega ,s\right)$ is given by the average over all possible directions φ (cf. Equation (22))

$$\hat{\tilde{h}}\left(\omega ,s\right)=\int_{0}^{2\pi }\beta \left(\varphi \right)\hat{\tilde{h}}\left(\omega ,s|\varphi \right)d\varphi ,$$

and a similar expression for $\hat{\tilde{H}}\left(\omega ,s\right)$.

### 4.2. The Isotropic and Uniform Case

In an isotropic medium (cf. Section 2.3) the pausing time densities are independent of the direction taken by the particle, ψ(t|φ)=ψ(t) and Ψ(t|φ)=Ψ(t), and for uniform motion we have [cf. Equations (27)–(29)]

(48) h(r,t|φ)=δ(r−ctu)ψ(t),H(r,t|φ)=δ(r−ctu)Ψ(t),

and the Fourier transforms are

(49) $$\tilde{h}\left(\omega ,t|\varphi \right)=\psi \left(t\right)e^{i(\omega \cdot u)ct},\;\;\tilde{H}\left(\omega ,t|\varphi \right)=\Psi \left(t\right)e^{i(\omega \cdot u)ct},$$

where u is the unit vector pointing in direction φ,

u=(cosφ,sinφ).

Assuming that all directions are equally likely (i.e., complete isotropy), we have

$$\beta \left(\varphi \right)=\frac{1}{2\pi }$$

and

$$\begin{array}{ccc} \tilde{h}\left(\omega ,t\right) & = & \int_{0}^{2\pi }b\left(\varphi \right)\tilde{h}\left(\omega ,t|\varphi \right)d\varphi =\frac{\psi (t)}{2\pi }\int_{0}^{2\pi }e^{ict|\omega |\cdot u}d\varphi \\ & = & \frac{\psi (t)}{2\pi }\int_{0}^{2\pi }e^{ict|\omega |\cos\varphi }d\varphi =\frac{\psi (t)}{\pi }\int_{0}^{\pi }\cos\left(ct|\omega |\cos\varphi \right)d\varphi . \end{array}$$

From the integral representation of the Bessel function $J_{0}\left(z\right)$ [63],

(50) $$J_{0}\left(ct|\omega |\right)=\frac{1}{\pi }\int_{0}^{\pi }\cos\left(ct|\omega |\cos\varphi \right)d\varphi ,$$

we get

(51) $$\tilde{h}\left(\omega ,t\right)=\psi \left(t\right)J_{0}\left(ct|\omega |\right),$$

and analogously

(52) $$\tilde{h}\left(\omega ,t\right)=\Psi \left(t\right)J_{0}\left(ct|\omega |\right).$$

For exponentially distributed sojourn intervals $\psi \left(t\right)=\lambda e^{-\lambda t}$ and $\Psi \left(t\right)=e^{-\lambda t}$, we write

$$\tilde{h}\left(\omega ,t\right)=\lambda e^{-\lambda t}J_{0}\left(ct|\omega |\right),\;\;\tilde{H}\left(\omega ,t\right)=\frac{1}{\lambda }\tilde{h}\left(\omega ,t\right).$$

Using the Laplace transformation formula [58]

$$L\left\{J_{0}\left(ct|\omega |\right)\right\}=\frac{1}{\sqrt{s^{2}+c^{2}t^{2}{\left|\omega \right|}^{2}}},$$

and the standard property

$$L\left\{e^{-\lambda t}f\left(t\right)\right\}=\hat{f}\left(\lambda +s\right),$$

we get

$$\hat{\tilde{h}}\left(\omega ,s\right)=\frac{\lambda }{\sqrt{{\left(\lambda +s\right)}^{2}+c^{2}{\left|\omega \right|}^{2}}}$$

and $\hat{\tilde{h}}\left(\omega ,s\right)=\hat{\tilde{h}}\left(\omega ,s\right)/\lambda$. Finally, from the Montroll–Weiss Equation (47) we obtain the exact solution to the homogeneous and isotropic random walk on the plane,

(53) $$\hat{\tilde{p}}\left(\omega ,s\right)=\frac{1}{\sqrt{{\left(\lambda +s\right)}^{2}+c^{2}{\left|\omega \right|}^{2}}-\lambda }.$$

Notice the completely different form for the exact PDF of the planar model compared to that of the three dimensional case given by Equation (35).

### 4.3. Fluid Limit Approximation and Telegrapher’s Equation

As we have done in the three dimensional transport, in order to get the two-dimensional equation we first make the fluid limit approximation of the planar model, that is, the long-distance and long-time limits of the exact PDF (53). By mimicking the steps done in Section 3.1 to obtain the fluid limit approximation in the three dimensional case, we can easily see that in two dimensions we obtain the same result but with the replacement

(54) $$c^{2}/6⟶c^{2}/4.$$

Thus, the approximation for the PDF reads (cf. Equation (41))

(55) $$\hat{\tilde{p}}\left(\omega ,s\right)=\frac{s+\lambda /2}{s\left(s+\lambda /2\right)+c^{2}{\left|\omega \right|}^{2}/4},$$

and similar expressions for the quantities $\hat{\tilde{h}}$ and $\hat{\tilde{H}}$. Assuming the initial conditions given in Equation (42), inverting Equation (55) and following the same procedure as in the three dimensional case we finally obtain the two-dimensional TE,

(56) $$\frac{\partial^{2}p}{\partial t^{2}}+\frac{1}{\tau }\frac{\partial p}{\partial t}=v^{2}\nabla^{2}p,$$

where as in three dimensions $\tau ={\left(2\lambda \right)}^{-1}$ but now

v=c/2.

Before proceeding further and explaining the fractional generalizations of telegraphic transport, let us point the significant issue of boundary conditions which are instrumental in first-passage, escape and survival problems. The question is far from being simple specially for telegraphic processes and in one and higher dimensions it has, to my knowledge, not being settled yet. In the transport of particles the problem of survival is closely related to the question of when the particle is absorbed (and, hence, disappears) if it reaches a certain critical region of boundary $S_{c}$. For diffusion processes, absorption at $S_{c}$ corresponds to $p(r,t|r_{0})=0$ when $r\in S_{c}$ (or $p(r,t|r_{0})=0$ when $r_{0}\in S_{c}$). That is, if the particle reaches $S_{c}$ (or starts at $S_{c}$) disappears. For telegraphic processes (and in the context of particle transport, at least for one-dimensional processes) the situation is more complex because of the property of persistence inherent in the telegrapher’s equation [14]. In this context persistence, which is analogous to the physical property of momentum, makes necessary, in deriving boundary conditions for absorption, to take into account the direction in which the particle is traveling. For if the particle starts at $S_{c}$, or at time *t* reaches $S_{c}$, will disappear (that is, it will be absorbed) only if the direction of the velocity is the appropriate one, otherwise the particle will escape.

For one dimensional processes we studied this situation some years ago [64,65] and refer the interested reader to these works for more information. In higher dimensions the situation may be even more involved. There are, however, problems which are not related to the escape out of some region (which implies absorption at the boundary of the region) but only on the first arrival to some region $S_{c}$. It can be shown that in these cases the boundary condition is $p(r,t|r_{0})=0$ (if $r\in S_{c}$ or $r_{0}\in S_{c}$), regardless the direction of the velocity at this particular location (see [66] for a problem of this sort in one dimension).

## 5. Fractional Transport

Likewise the one dimensional case, the two and three dimensional telegraphic transport processes described above are ordinary (i.e., non-fractional) processes in the sense that for small time (t≪τ) they behave like an ordinary wave front while for long times (t≫τ) they act like an ordinary diffusion processes,

$${\langle |r\left(t\right)|}^{2}\rangle ∼t^{2}{\;\left(t\to 0\right),\;\;\langle |r\left(t\right)|}^{2}\rangle ∼t\;\left(t\to \infty \right).$$

However, in transport through highly disordered systems as, for instance, random media or fractal structures, ordinary diffusion becomes anomalous, that is

$${\langle |r\left(t\right)|}^{2}\rangle ∼t^{\alpha },$$

where α is any positive real number. Two questions arise: (i) How does this circumstance affect telegraphic transport? and (ii) what is the fractional TE ruling such kind of processes? In one dimension we addressed these questions by setting a fractional version of the persistent random walk on the line [32]. In higher dimensions we followed this path and generalized the continuous and isotropic walks described in previous sections [30,31]. Let us review these findings.

### 5.1. The Fractional Isotropic Walk

We will first work on the three dimensional case and obtain a fractional version of the homogenous and isotropic random walk in the fluid limit approximation. To this end we generalize the expressions of $\hat{\tilde{h}}\left(\omega ,s\right)$ and $\hat{\tilde{H}}\left(\omega ,s\right)$—given in Section 3.1 in the fluid limit approximation—to include fractional behavior.

Let us start with Equation (39) which when s→0 yields

$$1-\hat{\tilde{h}}\left(\omega ,s\right)=\frac{1}{{\left(\lambda +s\right)}^{3}}\left[\lambda^{2}s+2\lambda s+\frac{\lambda }{3}{\left|\omega \right|}^{2}c^{2}+O(s^{3}{,|\omega |}^{4})\right],$$

we further approximate the denominator by ${\left(\lambda +s\right)}^{3}=\lambda^{3}+O\left(s\right)$, so that

$$1-\hat{\tilde{h}}\left(\omega ,s\right)≃\frac{s}{\lambda }+2{\left(\frac{\lambda }{2}\right)}^{2}+\frac{1}{3\lambda^{2}}{\left|\omega \right|}^{2}c^{2}\cdots .$$

We thus take as a fluid limit approximation for the sojourn density $\hat{\tilde{h}}$ the expression

(57) $$\hat{\tilde{h}}\left(\omega ,s\right)≃1-\frac{s}{\lambda }-2{\left(\frac{s}{\lambda }\right)}^{2}-\frac{1}{3\lambda^{2}}{\left|\omega \right|}^{2}c^{2}\cdots$$

We next obtain an appropriate fluid limit approximation for the sojourn probability $\hat{\tilde{H}}$. Thus starting from Equation (34) and following the same approximation scheme we get

$$\begin{array}{ccc} \hat{\tilde{H}}\left(\omega ,s\right) & = & \frac{1}{{\left(\lambda +s\right)}^{3}}\left[{\left(\lambda +s\right)}^{2}-\frac{1}{3}{\left|\omega \right|}^{2}c^{2}+{O(|\omega |}^{4})\right]\\ & = & \frac{1}{{\left(\lambda +s\right)}^{3}}\left[\lambda^{2}+2\lambda s-\frac{1}{3}{\left|\omega \right|}^{2}c^{2}+O(s^{2}{,|\omega |}^{4})\right].\\ & ≃ & \frac{1}{\lambda^{3}}\left(\lambda^{2}+2\lambda s\right)\cdots , \end{array}$$

That is,

(58) $$\hat{\tilde{H}}\left(\omega ,s\right)=\frac{1}{\lambda }\left(1+\frac{2s}{\lambda }\right)\cdots$$

Let us incidentally note that substituting Equations (57) and (58) into Montroll–Weiss Equation (26) yields the fluid limit solution (41) for the PDF $\hat{\tilde{p}}\left(\omega ,s\right)$ which has been the starting point of the derivation of the TE.

We are now ready to construct a fractional generalization of the three-dimensional isotropic random walk. Thus, and looking at Equation (57), we propose the following expansion for the sojourn density in the fluid limit:

(59) $$\hat{\tilde{h}}\left(\omega ,s\right)=1-{\left(Ts\right)}^{\alpha }-2{\left(Ts\right)}^{2\alpha }-\frac{1}{3}{\left(L|\omega |\right)}^{2\gamma }\cdots$$

(s,|ω|→0), where 0<α≤1, 0<γ≤1 and T>0 and L>0 are arbitrary parameters, *T* defines a characteristic time and *L* a characteristic length.

In addition to the fractional approximation for $\hat{\tilde{h}}\left(\omega ,s\right)$ we also assume a fractional expansion for the function $\hat{\tilde{H}}\left(\omega ,s\right)$ consistent with Equation (59). To this end, we return to Section 2 and average Equations (11) and (12) over all directions Ω, with the result (in Laplace space)

(60) $$\int_{R^{3}}\hat{h}\left(r,s\right)d^{3}r=\hat{\psi }\left(s\right),\;\;\int_{R^{3}}\hat{H}\left(r,s\right)d^{3}r=\hat{\Psi }\left(s\right),$$

where the sojourn PDF’s independent of direction are

$$\hat{h}\left(r,s\right)=\int \hat{h}\left(r,s|\Omega \right)\beta \left(\Omega \right)d^{2}\Omega ,\;\mathrm{and}\;\hat{\psi }\left(s\right)=\int \hat{\psi }\left(s|\Omega \right)\beta \left(\Omega \right)d^{2}\Omega ,$$

and similar expressions for $\hat{H}\left(r,s\right)$ and $\hat{\Psi }\left(s\right)$. Note that in terms of the Fourier transform we may write

$$\hat{\tilde{h}}\left(\omega =0,s\right)=\hat{\psi }\left(s\right),\;\mathrm{and}\;\hat{\tilde{H}}\left(\omega =0,s\right)=\hat{\Psi }\left(s\right).$$

However, Laplace transforming Equation (10) we see that $\hat{\Psi }\left(s\right)=\left[1-\hat{\psi }\left(s\right)\right]/s$, consequently,

$$\hat{\tilde{H}}\left(\omega =0,s\right)=\frac{1}{s}\left[1-\hat{\tilde{h}}\left(\omega =0,s\right)\right].$$

Inserting Equation (59) into this expression yields

$$\hat{\tilde{H}}\left(\omega =0,s\right)=T^{\alpha }s^{\alpha -1}+2T^{2\alpha }s^{2\alpha -1},$$

which leads us to conjecture the following fluid limit approximation:

(61) $$\hat{\tilde{H}}\left(\omega ,s\right)≃T^{\alpha }s^{\alpha -1}+2T^{2\alpha }s^{2\alpha -1}\cdots$$

(s→0,|ω|→0). Let us stress that this is simply a conjecture because the approximation given by Equation (61) might have depended on |ω| as well [32].

Substituting Equations (59) and (61) into Montroll–Weiss Equation (26) and reorganizing terms yields

$$\hat{\tilde{p}}\left(\omega ,s\right)=\frac{2T^{2\alpha }s^{\alpha -1}\left[s^{\alpha }+1/2T^{\alpha }\right]}{2T^{2\alpha }[s^{2\alpha }+s^{\alpha }/2T^{\alpha }+{\left|\omega \right|}^{2\gamma }\left(L^{2\gamma }/6T^{2\alpha }\right)]},$$

that is,

(62) $$\hat{\tilde{p}}\left(\omega ,s\right)=\frac{s^{\alpha -1}\left(s^{\alpha }+1/\tau \right)}{s^{2\alpha }+s^{\alpha }/\tau +v^{2}{\left|\omega \right|}^{2\gamma }},$$

where

(63) $$\tau =2T^{\alpha },\;v=\frac{1}{\sqrt{6}}\left(L^{\gamma }/T^{\alpha }\right),$$

(0<α≤1,0<γ≤1). The parameters τ and *v* can be considered as a fractional time and a fractional characteristic velocity, respectively.

### 5.2. Fractional Telegrapher’s Equation in Three Dimensions

To derive the fractional telegrapher’s equation (FTE) in three dimensions for the fractional isotropic model we first need to introduce some mathematical formalism concerning fractional derivatives.

The Caputo fractional derivative of order β>0 of a function ϕ(t) is defined by the functional [21,52,53,67,68]

(64) $$\frac{\partial^{\beta }\phi \left(t\right)}{\partial t^{\beta }}=\left\{\begin{array}{cc} \frac{1}{\Gamma (n-\beta )}\int_{0}^{t}\frac{\phi^{\left(n\right)}\left(t^{\prime }\right)dt^{\prime }}{{\left(t-t^{\prime }\right)}^{1+\beta -n}}, & n-1<\beta <n,\\ \phi^{\left(n\right)}\left(t\right), & \beta =n, \end{array}\right.$$

(n=1,2,3,⋯). Using this definition we can readily obtain the Laplace transform of the Caputo derivative. Indeed, Laplace transforming Equation (64) and using the convolution theorem we obtain

$$L\left\{\frac{\partial^{\beta }\phi \left(t\right)}{\partial t^{\beta }}\right\}=\frac{1}{\Gamma (n-\beta )}L\left\{\phi^{\left(n\right)}\left(t\right)\right\}L\left\{t^{n-\beta -1}\right\},$$

where L{·} stands for the Laplace transform. With the explicit forms [58]

$$L\left\{\phi^{\left(n\right)}\left(t\right)\right\}=s^{n}\hat{\phi }\left(s\right)-\sum_{k=0}^{n-1}s^{n-1-k}\phi^{\left(k\right)}\left(0\right),$$

and

$$L\left\{t^{n-\beta -1}\right\}=\Gamma \left(n-\beta \right)s^{\beta -n},$$

the Laplace transform of the Caputo derivative is found to be

(65) $$L\left\{\frac{\partial^{\beta }\phi \left(t\right)}{\partial t^{\beta }}\right\}=s^{\beta }\hat{\phi }\left(s\right)-s^{\beta -1}\phi \left(0\right)-\sum_{k=1}^{n-1}s^{\beta -1-k}\phi^{\left(k\right)}\left(0\right).$$

The second kind of fractional operator we need is the Riesz–Feller fractional Laplacian of order β (0<β≤2) of a function g(x) vanishing at x→±∞. There are several equivalent ways to define it [68], although one of the simplest and most operative definitions is obtained using Fourier analysis. We thus define [21]:

(66) $$\nabla^{\beta }g\left(r\right)=F^{-1}\left\{-{\left|\omega \right|}^{\beta }\tilde{g}\left(\omega \right)\right\},$$

(0<β≤2), where $F^{-1}\left\{\cdot \right\}$ stands for the inverse Fourier transform, and

$$\tilde{g}\left(\omega \right)=\int_{R^{3}}e^{i\omega \cdot r}g\left(r\right)d^{3}r,$$

is the direct transform.

We are now ready to derive the three-dimensional FTE. We begin with Equation (62) which we rewrite as

$$\left(s^{2\alpha }+\frac{1}{\tau }s^{\alpha }+v^{2}{\left|\omega \right|}^{2\gamma }\right)\hat{\tilde{p}}\left(\omega ,s\right)=s^{2\alpha -1}+\frac{1}{\tau }s^{\alpha -1}.$$

Taking into account the definition of the Riesz–Feller Laplacian, Equation (66), and recalling that $F^{-1}\left\{1\right\}=\delta \left(r\right)$, the Fourier inversion yields

$$\left(s^{2\alpha }+\frac{1}{\tau }s^{\alpha }-v^{2}\nabla^{2\gamma }\right)\hat{p}\left(r,s\right)=\left(s^{2\alpha -1}+\frac{1}{\tau }s^{\alpha -1}\right)\delta \left(r\right),$$

and, after reorganizing terms, we have

(67) $$s^{2\alpha }\hat{p}\left(r,s\right)-s^{2\alpha -1}\delta \left(r\right)+\frac{1}{\tau }\left[s^{\alpha }\hat{p}\left(r,s\right)-s^{\alpha -1}\delta \left(r\right)\right]=v^{2}\nabla^{2\gamma }\hat{p}.$$

In order to Laplace invert this equation, and thus obtaining an equation for p(r,t), we first evaluate tle Laplace transforms of the fractional derivatives $\partial^{\alpha }p/\partial^{\alpha }p$ and $\partial^{2\alpha }p/\partial^{2\alpha }p$ using Equation (65). We must distinguish the cases β=α and β=2α.

(i) Set β=α in Equation (65). Since 0<α≤1, we see that n=1. Hence

$$L\left\{\frac{\partial^{\alpha }p\left(r,t\right)}{\partial t^{\alpha }}\right\}=s^{\alpha }\hat{p}\left(r,s\right)-s^{\alpha -1}p\left(r,0\right).$$

However, p(r,0)=δ(r) (cf. Equation (42)). Therefore

(68) $$\frac{\partial^{\alpha }p\left(r,t\right)}{\partial t^{\alpha }}=L^{-1}\left\{s^{\alpha }\hat{p}\left(r,s\right)-s^{\alpha -1}\delta \left(r\right)\right\}.$$

(ii) When β=2α(0<α≤1) we need to distinguish the cases (a) 0<α≤1/2 and (b) 1/2<α≤1. For case (a) we have 0<2α≤1, which reproduces the conditions leading to Equation (68), That is,

$$L\left\{\frac{\partial^{2\alpha }p\left(r,t\right)}{\partial t^{2\alpha }}\right\}=s^{2\alpha }\hat{p}\left(r,s\right)-s^{2\alpha -1}\delta \left(r\right).$$

In case (b) we have 1<2α≤2 and from Equation (65) with n=2 we write

$$L\left\{\frac{\partial^{2\alpha }p\left(r,t\right)}{\partial t{}^{2}\alpha }\right\}=s^{2\alpha }\hat{p}\left(r,s\right)-s^{2\alpha -1}\delta \left(r\right)-s^{2(\alpha -1)}{\left.\frac{\partial p(r,t)}{\partial t}\right|}_{t=0}.$$

Since ${\partial p/\partial t|}_{t=0}=0$ (cf. Equation (42)) we see that this case coincides with case (a) above. Therefore,

(69) $$\frac{\partial^{2\alpha }p}{\partial t^{2\alpha }}=L^{-1}\left\{s^{2\alpha }\hat{p}\left(r,s\right)-s^{2\alpha -1}\delta \left(r\right)\right\},$$

(0<α≤1). Returning to Equation (67) and taking the inverse transform we find

$$L^{-1}\left\{s^{2\alpha }\hat{p}\left(r,s\right)-s^{2\alpha -1}\delta \left(r\right)\right\}+2\lambda L^{-1}\left\{s^{\alpha }\hat{p}\left(r,s\right)-s^{\alpha -1}\delta \left(r\right)\right\}=v^{2}\nabla^{2\gamma }p.$$

Using Equations (68) and (69), we readily obtain

(70) $$\frac{\partial^{2\alpha }p}{\partial t^{2\alpha }}+\frac{1}{\tau }\frac{\partial^{\alpha }p}{\partial t^{\alpha }}=v^{2}\nabla^{2\gamma }p,$$

which is the fractional telegrapher’s equation in three dimensions where τ is the fractional time and *v* the fractional velocity (cf. Equation (63)).

As is well known, and as we have remarked in previous sections, the ordinary TE enjoys both wave and diffusion characteristics. We now extend this duality to the fractional TE. To this end we take the limit τ→0 in Equation (70) and also letting v→∞ such that $\tau v^{2}\to D$ finite. This results in the fractional diffusion equation (cf. Equation (7))

(71) $$\frac{\partial^{\alpha }p}{\partial t^{\alpha }}=D\nabla^{2\gamma }p.$$

Let us see that for any values of τ and *v* the fractional diffusion equation is also the asymptotic (in time) limit of the fractional TE (recall that a similar situation occurs with the ordinary TE). Indeed, by passing to the limit s→0 in the fluid-limit expression of the PDF (cf. Equation (62)) the small *s* approximation for $\hat{\tilde{p}}\left(\omega ,s\right)$ is readily found to be

$$\hat{\tilde{p}}\left(\omega ,s\right)≃\frac{s^{\alpha -1}}{s^{\alpha }+\left(\tau v^{2}\right){\left|\omega \right|}^{2\gamma }},$$

which after Fourier-Laplace inversion yields Equation (71) with $D=\tau v^{2}$. Therefore, by virtue of Tauberian theorems the fractional diffusion Equation (71) is the long-time approximation of the fractional TE.

The fractional TE also contains the fractional wave equation as a special case. Thus letting τ→∞ with *v* finite in Equation (70) we get

(72) $$\frac{\partial^{2\alpha }p}{\partial t^{2\alpha }}=v^{2}\nabla^{2\gamma }p.$$

Note that when α=1/2 and γ=1 this equation reduces to the ordinary diffusion equation. In this regard Mainardi’s terminology “fractional diffusion-wave equation” [52] is more precise than “fractional wave equation”. Let us finally observe that the fractional diffusion-wave equation is the small-time limit of the fractional TE. Indeed, the limit s→∞ in Equation (62) yields

$$\hat{\tilde{p}}\left(\omega ,s\right)≃\frac{s^{2\alpha -1}}{s^{2\alpha }+v^{2}{\left|\omega \right|}^{2\gamma }},$$

and the Fourier–Laplace inversion results in Equation (72). Again, due to Tauberian theorems we see that the fractional diffusion-wave equation is the short-time limit of the fractional TE.

We thus see from the preceding discussion that the fractional TE embraces two different dynamics. one of them, at small times, representing fractional wavelike behavior, and another one which at long times enhances fractional diffusion-like behavior. This constitutes the fractional generalization of the dual character between waves and diffusions showed by the ordinary TE.

### 5.3. Lower Dimensional Cases

We next address lower dimensional problems and will see that in one and two dimensions the fractional TE has formally the same form than in three dimensions.

#### 5.3.1. One Dimension

As we know the one-dimensional fractional case is based the fractional generalization of the continuous-time persistent random walk on the line which is a discrete two-state model and whose derivation from the continuous multistate model described above, although similar in many aspects, it is not straightforward. We will here state just the main result and refer the interested reader to [32] or the review [15] for details. Thus, the one-dimensional fractional TE has formally the same appearance that the three-dimensional Equation (70)

(73) $$\frac{\partial^{2\alpha }p}{\partial t^{2\alpha }}+\frac{1}{\tau }\frac{\partial^{\alpha }p}{\partial t^{\alpha }}=v^{2}\frac{\partial^{2\gamma }p}{\partial x^{2\gamma }},$$

where the Caputo derivatives with respect to time are equal to those of the three dimensional Equation (70) and

$$\frac{\partial^{2\gamma }p}{\partial x^{2\gamma }}=F^{-1}\left\{-{\left|\omega \right|}^{2\gamma }\tilde{p}\left(\omega ,t\right)\right\},$$

is the Riesz–Feller fractional derivative (cf. Equation (66)), where

$$\tilde{p}\left(\omega ,t\right)=\int_{-\infty }^{\infty }e^{i\omega x}p\left(x,t\right)dx,$$

is the Fourier transform of the one-dimensional PDF p(x,t) and $F^{-1}\left\{\cdot \right\}$ is the inverse transform.

As the reader can easily check, the solution of Equation (73) with the standard initial conditions:

$$p\left(x,0\right)=\delta \left(x\right),\;{\left.\frac{\partial p(x,t)}{\partial t}\right|}_{t=0}=0,$$

in Fourier–Laplace space reads

(74) $$\hat{\tilde{p}}\left(\omega ,s\right)=\frac{s^{\alpha -1}\left(s^{\alpha }+1/\tau \right)}{s^{2\alpha }+s^{\alpha }/\tau +v^{2}{\left|\omega \right|}^{2\gamma }}.$$

which is the one-dimensional version of Equation (62).

#### 5.3.2. Two Dimensions

As we have discussed in Section 4.3 most expressions of the two-dimensional case are the same than in three dimensions after the replacement $c^{2}/6\to c^{2}/4$ (cf. Equation (54)). Thus, for instance, the sojourn densities $\hat{\tilde{h}}$ and $\hat{\tilde{H}}$—which in three dimensions and in the fluid limit approximations are given by Equations (57) and (58)—now read

(75) $$\hat{\tilde{h}}\left(\omega ,s\right)≃1-\frac{s}{\lambda }-2{\left(\frac{s}{\lambda }\right)}^{2}-\frac{1}{2\lambda^{2}}{\left|\omega \right|}^{2}c^{2}\cdots ,\;\hat{\tilde{H}}\left(\omega ,s\right)=\frac{1}{\lambda }\left(1+\frac{2s}{\lambda }\right)\cdots$$

(s→0,|ω|→0) which mimicking the discussion of Section 5.1 leads to the following fractional generalization of these functions [see Equations (59) and (61)]

(76) $$\hat{\tilde{h}}\left(\omega ,s\right)=1-{\left(Ts\right)}^{\alpha }-2{\left(Ts\right)}^{2\alpha }-\frac{1}{2}{\left(L|\omega |\right)}^{2\gamma }\cdots ,\;\hat{\tilde{H}}\left(\omega ,s\right)≃T^{\alpha }s^{\alpha -1}+2T^{2\alpha }s^{2\alpha -1}\cdots$$

Substituting Equation (76) into Montroll–Weiss Equation (26) and reorganizing terms yields

(77) $$\hat{\tilde{p}}\left(\omega ,s\right)=\frac{s^{\alpha -1}\left(s^{\alpha }+1/\tau \right)}{s^{2\alpha }+s^{\alpha }/\tau +v^{2}{\left|\omega \right|}^{2\gamma }},$$

where

(78) $$\tau =2T^{\alpha },\;v=\frac{1}{\sqrt{2}}\left(L^{\gamma }/T^{\alpha }\right).$$

Equation (77) gives the transformed PDF of the fractional two-dimensional isotropic random walk in the fluid limit approximation. Let us note that this expression has the same form as that of the three-dimensional case (cf. Equation (62)) with the same time parameter τ but a different velocity parameter *v* (cf. Equation (63)). Therefore, following exactly the same procedure detailed in the previous section we find the two-dimensional TE

(79) $$\frac{\partial^{2\alpha }p}{\partial t^{2\alpha }}+\frac{1}{\tau }\frac{\partial^{\alpha }p}{\partial t^{\alpha }}=v^{2}\nabla^{2\gamma }p,$$

which has the same form as the fractional TE (70) of the three dimensional case and with the same limiting behavior regarding fractional diffusion and wave-like performances.

### 5.4. Characteristic Function

Solving the fractional telegrapher’s equation and thus obtaining the exact analytical form of the PDF p(r,t) seems to be out of reach for any dimension. It is, however, possible to obtain regardless dimensionality, a close and exact expression of the characteristic function $\tilde{p}\left(\omega ,t\right)$ (i.e., the Fourier transform of the PDF p(r,t)) of the space-time fractional telegrapher’s Equation (70). To this end we will perform the Laplace inversion of the joint Fourier–Laplace $\hat{\tilde{p}}\left(\omega ,s\right)$. Since this function has formally the same form in one, two and tree dimensions (cf. Equations (74), (77) and (62) respectively) the differences between them only arise when Fourier inverting and the characteristic function will be formally the same in all cases. This similarity also shows that the time structure of any average will be the same regardless the number of spatial dimension (we will see this fact explicitly in our discussion on the moments of time-fractional processes to be discussed in the next section).

We start off with Equation (77). Let us first note that taking into account the factorization

$$s^{2\alpha }+s^{\alpha }/\tau +v^{2}{\left|\omega \right|}^{2\gamma }=\left[s^{\alpha }+\frac{1}{2\tau }-\eta \left(\omega \right)\right]\left[s^{\alpha }+\frac{1}{2\tau }+\eta \left(\omega \right)\right],$$

where

(80) $$\eta \left(\omega \right)=\sqrt{1/4\tau^{2}-v^{2}{\left|\omega \right|}^{2\gamma }},$$

Equation (77) can be written as

(81) $$\hat{\tilde{p}}\left(\omega ,s\right)=\frac{s^{\alpha -1}}{2\eta (\omega )}\left[\frac{1/2\tau +\eta (\omega )}{s^{\alpha }+1/2\tau -\eta \left(\omega \right)}-\frac{1/2\tau -\eta (\omega )}{s^{\alpha }+1/2\tau +\eta \left(\omega \right)}\right].$$

Further manipulations yield

$$\frac{s^{\alpha -1}}{s^{\alpha }+1/2\tau \pm \eta \left(\omega \right)}=\frac{s^{-1}}{1+\left[1/2\tau \pm \eta \left(\omega \right)\right]s^{-\alpha }}=\sum_{n=0}^{\infty }{\left(-1\right)}^{n}{\left[1/2\tau \pm \eta (\omega )\right]}^{n}s^{-1-n\alpha }.$$

We next proceed to Laplace inversion. Since [58]

$$L^{-1}\left\{s^{-1-n\alpha }\right\}=\frac{t^{n\alpha }}{\Gamma (1+n\alpha )},$$

we have

$$L^{-1}\left\{\frac{s^{\alpha -1}}{s^{\alpha }+1/2\tau \pm \eta \left(\omega \right)}\right\}=\sum_{n=0}^{\infty }{\left(-1\right)}^{n}\frac{{\left(\left[1/2\tau \pm \eta \left(\omega \right)\right]t^{\alpha }\right)}^{n}}{\Gamma (1+n\alpha )}=E_{\alpha }\left(-\left[1/2\tau \pm \eta \left(\omega \right)\right]t^{\alpha }\right),$$

where $E_{\alpha }\left(\cdot \right)$ is the Mittag–Leffler function [69]

(82) $$E_{\alpha }\left(z\right)=\sum_{n=0}^{\infty }\frac{z^{n}}{\Gamma (1+n\alpha )}.$$

For α not an integer, the Mittag–Leffler function $E_{\alpha }\left(z\right)$ can be regarded as a kind of “fractional generalization” of the exponential function. Indeed when α=1 and since Γ(1+n)=n! we immediately see from Equation (82) that $E_{1}\left(z\right)=e^{z}$.

Taking the inverse Laplace transform of Equation (81) and using the above intermediate results we finally obtain the characteristic function of the space-time fractional transport process

(83) $$\begin{array}{ccc} \tilde{p}\left(\omega ,t\right)=\frac{1}{2\eta (\omega )}\{[1/2\tau & + & \eta \left(\omega \right)]E_{\alpha }\left(-\left[1/2\tau -\eta \left(\omega \right)\right]t^{\alpha }\right)\\ & - & \left[1/2\tau -\eta (\omega )\right]E_{\alpha }\left(-\left[1/2\tau +\eta \left(\omega \right)\right]t^{\alpha }\right)\}, \end{array}$$

which we recall is valid for any dimension of the underlying space.

In the wave-like limit when *v* is finite but τ→∞ the fractional TE (70) reduces to the fractional wave-diffusion Equation (72). In this case (cf. Equation (80))

$$\eta \left(\omega \right)={iv|\omega |}^{\gamma },$$

and the characteristic function reads

(84) $$\tilde{p}\left(\omega ,t\right)=\frac{1}{2}\left[E_{\alpha }\left(-{iv|\omega |}^{\gamma }t^{\alpha }\right)+E_{\alpha }\left({iv|\omega |}^{\gamma }t^{\alpha }\right)\right],$$

a solution already obtained by Mainardi for the wave-diffusion equation [52].

In the diffusion-like limit τ→0 and v→∞ such that $2\tau v^{2}=D$ (finite) and from Equation (80) we see that

$$2\tau \eta \left(\omega \right)=\sqrt{1-4\tau^{2}v^{2}{\left|\omega \right|}^{2\gamma }}⟶1,$$

and

$$\frac{1}{2\tau }\mp \eta \left(\omega \right)=\frac{1}{2\tau }\left(1\mp \sqrt{1-{2\tau D|\omega |}^{2\gamma }}\right)=\frac{{D|\omega |}^{2\gamma }}{1\pm \sqrt{1-{2\tau D|\omega |}^{2\gamma }}}⟶D{\left|\omega \right|}^{2\gamma }.$$

From Equation (83) we get

(85) $$\tilde{p}\left(\omega ,t\right)=E_{\alpha }\left(-Dt^{\alpha }{\left|\omega \right|}^{2\gamma }\right),$$

a well known result which corresponds to a Levy density with fractional time [36,38]. When α=1 this result reduces to the ordinary Levy distribution with zero mean,

(86) $$\tilde{p}\left(\omega ,t\right)=e^{-{Dt|\omega |}^{2\gamma }}.$$

## 6. Time-Fractional Telegraphic Transport

In the last section we have developed the fractional telegraphic transport in its most general form assuming that both time and space are fractional. This leads, as the master equation of the process, to the space-time fractional telegrapher’s equation which is formally the same in one, two and three dimensions. We have also seen that in both cases the general space-time fractional TE reduces to the space-time fractional wave equation at short times and to the space-time fractional diffusion equation at long times. This dual character is even more manifest for the time-fractional equation when the spatial exponent γ=1 and only time is fractional. For fractional diffusion this particular case has been extensively studied in the literature and has many applications [35,36,37,39,41,42,43].

In any dimension the time-fractional TE is

(87) $$\frac{\partial^{2\alpha }p}{\partial t^{2\alpha }}+\frac{1}{\tau }\frac{\partial^{\alpha }p}{\partial t^{\alpha }}=v^{2}\nabla^{2}p$$

(0<α≤1), where $\nabla^{2}$ is either the two or the three dimensional Laplacian and in one dimension is the second spacial derivative.

For the time-fractional TE we can obtain more analytical results than for the space-time TE. Results that turn out to be very useful because they clearly mark the similarities and dissimilarities between telegraphic transport processes in different dimensions. For one dimension we had already obtained in [32] some of the results presented here but not in higher dimensions. We now fill this gap in which the analogies and differences among different dimensions are clearly manifested.

Our starting point is again the Fourier–Laplace solution of the fractional TE (cf. Equations (62), (74) or (77) with γ=1)

(88) $$\hat{\tilde{p}}\left(\omega ,s\right)=\frac{s^{\alpha -1}\left(s^{\alpha }+1/\tau \right)}{s^{2\alpha }+s^{\alpha }/\tau +v^{2}{\left|\omega \right|}^{2}}.$$

The basic idea is the following: since time is now the only fractional variable but not space, it is possible to Fourier invert $\hat{\tilde{p}}\left(\omega ,s\right)$ and obtain a closed expression for the Laplace transform $\hat{p}\left(r,s\right)$ which compels us to treat different dimensions separately. Once we get the expression for $\hat{p}\left(r,s\right)$, the use of Tauberian theorems will allow us to obtain asymptotic expressions of the PDF p(r,t) at long and short times. Even though the one dimensional problem was fully addressed in [32], we present here all three dimensions and compare each result.

### 6.1. Laplace Transform of the PDF

Let us proceed to Fourier invert the expression (88) for $\hat{\tilde{p}}\left(\omega ,s\right)$. To this end we need to treat each dimension separately.

#### 6.1.1. One Dimension

Recall that in one dimension the expression (88) of the transformed density $\hat{\tilde{p}}$ remains valid although in this case |ω| is not the modulus of a vector but the absolute value of a single variable. By virtue of the symmetry of $\hat{\tilde{p}}$ with respect to ω, the Fourier inversion will be given by

$$\hat{p}\left(x,s\right)=\frac{1}{2\pi }\int_{-\infty }^{\infty }e^{-i\omega x}\hat{\tilde{p}}\left(\omega ,s\right)d\omega =\frac{1}{\pi }\int_{0}^{\infty }\hat{\tilde{p}}\left(\omega ,s\right)\cos\omega xd\omega .$$

Substituting for Equation (88) yields

$$\hat{p}\left(x,s\right)=\frac{1}{\pi s}(s^{2\alpha }+s^{\alpha }/\tau )\int_{0}^{\infty }\frac{\cos\omega x}{s^{2\alpha }+s^{\alpha }/\tau +v^{2}\omega^{2}}d\omega ,$$

and recalling the integral [70]

$$\int_{0}^{\infty }\frac{\cos\omega x}{a^{2}+b^{2}\omega^{2}}d\omega =\frac{1}{2ab}e^{-a|x|/b},$$

we get

(89) $$\hat{p}\left(x,s\right)=\frac{1}{2vs}\sqrt{s^{2\alpha }+s^{\alpha }/\tau }\exp\left\{-\frac{\left|x\right|}{v}\sqrt{s^{2\alpha }+s^{\alpha }/\tau }\right\}.$$

For α=1 the fractional TE (87) reduces to the the ordinary TE and in this one-dimensional case the Laplace transform (89) can be inverted yielding the exact PDF p(x,t) in terms of modified Bessel functions. We refer the interested reader to [32] for more details. For the fractional case when α≠1, the exact analytical inversion of Equation (89) seems to be out of reach. However, as we will see below, we can obtain approximate solutions for large values of time.

#### 6.1.2. Two Dimensions

In this case the Fourier inversion formula yields for the PDF in two dimensions

$$\begin{array}{ccc} \hat{p}\left(r,s\right) & = & \frac{1}{{\left(2\pi \right)}^{2}}\int_{R^{2}}e^{-i\omega \cdot r}\hat{\tilde{p}}\left(\omega ,s\right)d^{2}\omega =\frac{1}{{\left(2\pi \right)}^{2}}\int_{0}^{\infty }\int_{0}^{2\pi }e^{-i\omega r\cos\varphi }\hat{\tilde{p}}\left(\omega ,s\right)\omega d\omega d\varphi \\ & = & \frac{1}{{\left(2\pi \right)}^{2}}\int_{0}^{\infty }\omega \hat{\tilde{p}}\left(\omega ,s\right)d\omega \int_{0}^{2\pi }e^{-i\omega r\cos\varphi }d\varphi , \end{array}$$

where ω=|ω| and we have taken into account that $\hat{\tilde{p}}\left(\omega ,s\right)$ depends only on the modulus |ω|=ω [see Equation (88)].

From the integral representation of the Bessel function of zero order [63],

$$J_{0}\left(\omega r\right)=\frac{1}{2\pi }\int_{0}^{2\pi }e^{-i\omega r\cos\varphi }d\varphi ,$$

we write

$$\hat{p}\left(r,s\right)=\frac{1}{2\pi }\int_{0}^{\infty }\omega J_{0}\left(\omega r\right)\hat{\tilde{p}}\left(\omega ,s\right)d\omega .$$

Substituting for (88) we have

$$\hat{p}\left(x,s\right)=\frac{1}{2\pi s}(s^{2\alpha }+s^{\alpha }/\tau )\int_{0}^{\infty }\frac{\omega J_{0}\left(\omega r\right)}{s^{2\alpha }+s^{\alpha }/\tau +v^{2}\omega^{2}}d\omega ,$$

and taking into account the integral [70]

$$\int_{0}^{\infty }\frac{\omega J_{0}\left(a\omega \right)}{b^{2}+\omega^{2}}d\omega =K_{0}\left(ab\right),$$

(a>0,Reb>0), where $K_{0}\left(\cdot \right)$ is a modified Bessel function, we finally obtain

(90) $$\hat{p}\left(r,s\right)=\frac{1}{2\pi v^{2}s}\left(s^{2\alpha }+s^{\alpha }/\tau \right)K_{0}\left(\frac{r}{v}\sqrt{s^{2\alpha }+s^{\alpha }/\tau }\right).$$

#### 6.1.3. Three Dimensions

Bearing in mind that $\hat{\tilde{p}}\left(\omega ,s\right)$ depends only on the modulus |ω|=ω (cf. Equation (88)), the Fourier inversion of $\hat{\tilde{p}}$ is

$$\begin{array}{ccc} \hat{p}\left(r,s\right) & = & \frac{1}{{\left(2\pi \right)}^{3}}\int_{R^{3}}e^{-i\omega \cdot r}\hat{\tilde{p}}\left(\omega ,s\right)d^{3}\omega \\ & = & \frac{1}{{\left(2\pi \right)}^{3}}\int_{0}^{\infty }\int_{0}^{\pi }\int_{0}^{2\pi }e^{-i\omega r\cos\theta }\hat{\tilde{p}}\left(\omega ,s\right)\omega^{2}\sin\theta d\omega d\theta d\varphi \\ & = & \frac{1}{{\left(2\pi \right)}^{2}}\int_{0}^{\infty }\omega^{2}\hat{\tilde{p}}\left(\omega ,s\right)d\omega \int_{0}^{\pi }e^{-i\omega r\cos\theta }\sin\theta d\theta . \end{array}$$

Since

$$\int_{0}^{\pi }e^{-i\omega r\cos\theta }\sin\theta d\theta =\frac{2}{\omega r}\sin\omega r,$$

we have

$$\hat{p}\left(r,s\right)=\frac{1}{2\pi^{2}r}\int_{0}^{\infty }\omega \sin\omega r\hat{\tilde{p}}\left(\omega ,s\right)d\omega .$$

Substituting for Equation (88) yields

$$\hat{p}\left(x,s\right)=\frac{1}{2\pi^{2}rs}(s^{2\alpha }+s^{\alpha }/\tau )\int_{0}^{\infty }\frac{\omega \sin\omega r}{s^{2\alpha }+s^{\alpha }/\tau +v^{2}\omega^{2}}d\omega ,$$

and taking into account the integral [70]

$$\int_{0}^{\infty }\frac{\omega \sin a\omega }{b^{2}+\omega^{2}}d\omega =\frac{\pi }{2}e^{-ab},$$

(a≥0,Reb>0), we obtain

(91) $$\hat{p}\left(r,s\right)=\frac{1}{4\pi rv^{2}s}\left(s^{2\alpha }+s^{\alpha }/\tau \right)\exp\left\{-\frac{r}{v}\sqrt{s^{2\alpha }+s^{\alpha }/\tau }\right\},$$

which is the exact PDF in three dimensions. Notice the different form taken by $\hat{p}\left(r,s\right)$ in one, two and three dimensions (cf. Equations (89)–(91), respectively).

Let us also observe that, like in the general space-time fractional cases, for the time-fractional case (α≠1, γ=1) the expressions for $\hat{p}$ given by Equations (89)–(91) are very difficult, not to say impossible, to invert analytically. Thus, obtaining the exact analytical form of the PDF p(r,t) in real time seems to be beyond reach. We can, however, obtain approximate solutions, valid for large values of time, using Tauberian theorems which relate the small *s* behavior of $\hat{p}\left(r,s\right)$ with the large *t* behavior of p(r,t) [61,71].

### 6.2. Long-Time Asymptotic Expressions

For the asymptotic analysis we rely on Tauberian theorems which allow us to infer the behavior of p(r,t) for long times out of the expression for $\hat{p}\left(r,s\right)$ for small values of the Laplace variable *s* [61,71]. We work again each dimension separately.

#### 6.2.1. One Dimension

We briefly summarize only the main results in one dimension and refer the reader to [32] for details. For long times such that $t\gg \tau^{1/\alpha }$ we have shown that [32]

(92) $$p\left(x,t\right)≃\frac{t^{-\alpha /2}}{2v\sqrt{\tau }}M_{\alpha /2}\left(\frac{\left|x\right|t^{-\alpha /2}}{2v\sqrt{\tau }}\right),\;\left(t\gg \tau^{1/\alpha }\right),$$

where $M_{\alpha /2}\left(\cdot \right)$ is the Mainardi function defined by the power series [52,72]

(93) $$M_{\beta }\left(z\right)=\sum_{n=0}^{\infty }\frac{{\left(-1\right)}^{n}z^{n}}{n!\Gamma (-\beta n+1-\beta )}.$$

The function $M_{\beta }\left(z\right)$ is an entire function for 0<β<1 [52] being a special case of the Wright function [69,72] (see below) which is, in turn, closely related to Fox function frequently used in the anomalous diffusion literature [36]. We incidentally note that after the replacement $v\sqrt{\tau }\to D$, the asymptotic expression (92) becomes the exact solution to the time fractional diffusion equation (cf. Equation (71) with γ=1), solution obtained by Mainardi some years ago [52].

From Equation (93) we see that $M_{\alpha /2}\left(z\right)\to 1/\Gamma \left(1-\alpha /2\right)$ as z→0 and Equation (92) yields the asymptotic power law

(94) $$p\left(x,t\right)∼t^{-\alpha /2},\;\left(t\to \infty \right).$$

#### 6.2.2. Two Dimensions

Noticing that as s→0 (specifically, if $s\ll \tau^{-1/\alpha }$)

(95) $$s^{2\alpha }+s^{\alpha }/\tau =\left(s^{\alpha }/\tau \right)\left(\tau s^{\alpha }+1\right)≃s^{\alpha }/\tau ,$$

we write for the two dimensional density (90)

(96) $$\hat{p}\left(r,s\right)≃\frac{s^{\alpha -1}}{2\pi v^{2}\tau }K_{0}\left(\frac{rs^{\alpha /2}}{v\sqrt{\tau }}\right),\;\;\left(s\ll \tau^{-1/\alpha }\right).$$

On the other hand [63]

$$K_{0}\left(z\right)=-\left[\gamma +\ln(z/2)\right]I_{0}\left(z\right)+2\sum_{n=1}^{\infty }\frac{1}{n}I_{2n}\left(z\right),$$

(γ=0.5772⋯ is the Euler constant and $I_{\nu }\left(z\right)$ are modified Bessel functions), but [63]

$$I_{\nu }\left(z\right)=\sum_{n=0}^{\infty }\frac{{\left(z/2\right)}^{\nu +2n}}{\Gamma (\nu +n+1)}=O\left(z^{\nu }\right)\;\left(z\to 0\right),$$

thus

(97) $$K_{0}\left(z\right)=-\left[\gamma +\ln(z/2)\right]+O\left(z^{2}\ln z\right).$$

Hence

$$K_{0}\left(\frac{rs^{\alpha /2}}{v\sqrt{\tau }}\right)=-\left[\gamma +\ln\left(\frac{r}{2v\sqrt{\tau }}\right)\right]-\frac{\alpha }{2}\ln s+O\left(s^{\alpha }\ln s\right),$$

which substituting into Equation (96) yields the approximate expressions valid for small values of *s* (i.e., when $s\ll \tau^{-1/\alpha }$)

(98) $$\hat{p}\left(r,s\right)≃\frac{-1}{2\pi v^{2}\tau }\left\{\left[\gamma +\ln\left(\frac{r}{2v\sqrt{\tau }}\right)\right]\frac{1}{s^{1-\alpha }}+\frac{\alpha /2}{s^{1-\alpha }}\ln s\right\}.$$

We next proceed to Laplace inverting this small *s* expression for $\hat{p}\left(r,s\right)$ which by virtue of Tauberian theorems will provide an approximate expression of p(r,t) suitable for long times. Taking into account the Laplace inversion formulae [32,58]

$$L\left\{\frac{1}{s^{\beta }}\right\}=\frac{t^{\beta -1}}{\Gamma (\beta )}\;\mathrm{and}\;L\left\{\frac{\ln s}{s^{\beta }}\right\}=\frac{t^{\beta -1}}{\Gamma (\beta )}\left[\psi (\beta )-\ln t\right]$$

[β>0 and $\psi \left(z\right)=\Gamma^{\prime }\left(z\right)/\Gamma \left(z\right)$] we have

$$\begin{array}{ccc} p(r,t) & ≃ & \frac{-1}{2\pi v^{2}\tau }\left\{\left[\gamma +\ln\left(\frac{r}{2v\sqrt{\tau }}\right)\right]\frac{t^{-\alpha }}{\Gamma (1-\alpha )}+\frac{\alpha }{2}\frac{t^{-\alpha }}{\Gamma (1-\alpha )}\left[\psi (1-\alpha )-\ln t\right]\right\}\\ & = & \frac{1}{2\pi v^{2}\tau }\frac{t^{-\alpha }}{\Gamma (1-\alpha )}\left[\frac{\alpha }{2}\ln t-\ln\left(\frac{r}{2v\sqrt{\tau }}\right)-\gamma -\psi \left(1-\alpha \right)\right]\\ & = & \frac{1}{2\pi v^{2}\tau }\frac{t^{-\alpha }}{\Gamma (1-\alpha )}\left[\ln\left(\frac{2vt^{\alpha /2}\sqrt{\tau }}{r}\right)-\gamma -\psi \left(1-\alpha \right)\right], \end{array}$$

and neglecting constant terms we finally get

(99) $$p\left(r,t\right)≃\frac{1}{2\pi v^{2}\tau }\frac{t^{-\alpha }}{\Gamma (1-\alpha )}\ln\left(\frac{2vt^{\alpha /2}\sqrt{\tau }}{r}\right),$$

($t\gg \tau^{1/\alpha }$). Therefore, in two dimensions the PDF of the time fractional TE obeys the asymptotic logarithmic power law

(100) $$p\left(x,t\right)∼t^{-\alpha }\ln t\;\left(t\to \infty \right).$$

#### 6.2.3. Three Dimensions

In three dimensions the starting point of our asymptotic analysis is Equation (91) which using the small *s* approximation given in Equation (95) yields

(101) $$\hat{p}\left(r,s\right)≃\frac{s^{\alpha -1}}{4\pi rv^{2}\tau }e^{-rs^{\alpha /2}/v\sqrt{\tau }}\;\left(s\ll \tau^{-1/\alpha }\right),$$

and expanding the exponential we write

$$\hat{p}\left(r,s\right)≃\frac{s^{\alpha -1}}{4\pi rv^{2}\tau }\sum_{n=0}^{\infty }\frac{1}{n!}{\left(\frac{-r}{v\sqrt{\tau }}\right)}^{n}s^{-1+(1+n/2)\alpha }\;\left(s\ll \tau^{-1/\alpha }\right).$$

Recall again that because of Tauberian theorems the inversion of this expression for $\hat{p}\left(r,s\right)$, valid for small values of *s*, will provide an asymptotic expression for p(r,t) suitable for large values of *t*. Thus, taking into account the Laplace inversion formula [32,52]

(102) $$L^{-1}\left\{s^{\delta }\right\}=\frac{t^{-1-\delta }}{\Gamma (-\delta )}$$

(where δ≠0 and not a positive integer) we obtain for $t\gg \tau^{1/\alpha }$

$$p\left(r,t\right)≃\frac{1}{4\pi rv^{2}\tau }\sum_{n=0}^{\infty }\frac{1}{n!}{\left(\frac{-r}{v\sqrt{\tau }}\right)}^{n}\frac{t^{-(1+n/2)\alpha }}{\Gamma [1-(1+n/2)\alpha ]},$$

that is

(103) $$p\left(r,t\right)≃\frac{t^{-\alpha }}{4\pi rv^{2}\tau }\sum_{n=0}^{\infty }\frac{1}{n!}\frac{1}{\Gamma [1-(1+n/2)\alpha ]}{\left(\frac{-rt^{-\alpha /2}}{v\sqrt{\tau }}\right)}^{n},\;\left(t\gg \tau^{1/\alpha }\right).$$

This asymptotic expression for p(r,t) can be written in a more compact form by using the Wright function defined by [69,72]

(104) $$W_{\lambda ,\mu }\left(z\right)=\sum_{n=0}^{\infty }\frac{z^{n}}{n!\Gamma (\mu +\lambda n)},$$

(λ>−1 and μ and *z* arbitrary complex numbers). It is an entire function originally proposed by Wright in the 1930’s for the asymptotic theory of partitions [69]. When λ=1 the Wright function can be written in terms of the Bessel function of order μ−1 [69,72]. Moreover, Mainardi function $M_{\beta }\left(z\right)$ defined in Equation (93) is a particular case of the Wright function. Indeed,

$$M_{\beta }\left(z\right)=W_{-\beta ,1-\beta }\left(-z\right).$$

From Equations (103) and (104) we see that the asymptotic PDF for the three dimensional case can be written as

(105) $$p\left(r,t\right)≃\frac{t^{-\alpha }}{4\pi rv^{2}\tau }W_{\alpha /2,1-\alpha }\left(\frac{-rt^{-\alpha /2}}{v\sqrt{\tau }}\right),\;\left(t\gg \tau^{1/\alpha }\right).$$

Finally, from Equation (104) we see that $W_{\lambda ,\mu }\left(z\right)\to 1/\Gamma \left(\mu \right)$ as z→0 and Equation (105) yields the asymptotic power law

(106) $$p\left(r,t\right)∼t^{-\alpha },\;\left(t\to \infty \right).$$

### 6.3. Moments of the Effective Distance Travelled

One of the magnitudes of greatest interest in transport problems is the distance covered by the particle from the starting point. The evaluation of the actual distance is very involved due to the random turnarounds of the trajectory. We will take as an estimate of it the effective distance travelled (taking into account that the transport processes starts at the origin of the coordinate system) which is the quantity |x(t)| in one dimension, and |r(t)|=r(t) in two and three dimensions. We will thus work each dimension separately, although, as stated in Section 5.4, moments are essentially the same for each dimension.

Let us note that for space-time fractional processes, the moments of the distance travelled may be infinite (as in the case of the Levy processes). However, for time-fractional processes these moments are finite and we can get analytical expressions for them using the forms of the PDF obtained above. Moments also make explicit the dual character of the fractional telegraphic transport between fractional wave transport and fractional diffusion transport which generalizes the same duality presented by the ordinary TE.

#### 6.3.1. One Dimension

Moments are defined by

$${\langle |x\left(t\right)|}^{n}\rangle =\int_{-\infty }^{\infty }{\left|x\right|}^{n}p\left(x,t\right),\;\left(n=1,2,\cdots \right),$$

and recalling that p(x,t) is an even function of *x*, the Laplace transform can be written as

$$L\left\{\langle |x\left(t\right){|}^{n}\rangle \right\}=2\int_{0}^{\infty }x^{n}\hat{p}\left(x,s\right)dx.$$

Substituting for Equation (89) yields

$$L\left\{\langle |x\left(t\right){|}^{n}\rangle \right\}=\frac{\sqrt{\beta (s)}}{vs}\int_{0}^{\infty }x^{n}e^{-x\sqrt{\beta (s)}/v}dx=\frac{v^{n}}{s{\left[\beta \left(s\right)\right]}^{n/2}}\int_{0}^{\infty }z^{n}e^{-z}dz=\frac{v^{n}n!}{s{\left[\beta \left(s\right)\right]}^{n/2}},$$

where $\beta \left(s\right)=s^{2\alpha }+s^{\alpha }/\tau$. Hence

(107) $$L\left\{\langle |x\left(t\right){|}^{n}\rangle \right\}=\frac{n!v^{n}}{s{\left(s^{2\alpha }+s^{\alpha }/\tau \right)}^{n/2}},\;\left(n=1,2,\cdots \right).$$

Recall that when τ→∞ we recover the fractional wave equation. In this case from Equation (107) we have the “wave limit”

(108) $$L\left\{\langle |x\left(t\right){|}^{n}\rangle \right\}=\frac{n!v^{n}}{s^{1+n\alpha }}\;\Rightarrow {\;\langle |x\left(t\right)|}^{n}\rangle =\frac{n!v^{n}}{\Gamma (1+n\alpha )}t^{n\alpha }.$$

On the other hand when τ→0 but v→∞ such that $v^{2}\tau =D$ (finite) we recover the fractional diffusion equation and have the “diffusion limit”

(109) $$L\left\{\langle |x\left(t\right){|}^{n}\rangle \right\}=\frac{n!D^{n/2}}{s^{1+n\alpha /2}}\;\Rightarrow {\;\langle |x\left(t\right)|}^{n}\rangle =\frac{n!D^{n/2}}{\Gamma (1+n\alpha /2)}t^{n\alpha /2}.$$

Let us also recall that the wave limit is the one we recover from TE as t→0, whereas the diffusion limit corresponds to the long time limit. Indeed, taking into account that

$${\left(s^{2\alpha }+s^{\alpha }/\tau \right)}^{n/2}≃s^{n\alpha }\;\left(s\to \infty \right)\;\mathrm{and}\;{\left(s^{2\alpha }+s^{\alpha }/\tau \right)}^{n/2}≃{\left(s^{\alpha }/\tau \right)}^{n}\;\left(s\to 0\right)$$

and bearing in mind Tauberian theorems, we see from Equation (107)

(110) $$L\left\{\langle |x\left(t\right){|}^{n}\rangle \right\}≃\frac{n!v^{n}}{s^{1+n\alpha }}\;\left(s\to \infty \right)\;\Rightarrow {\;\langle |x\left(t\right)|}^{n}\rangle ≃\frac{n!v^{n}}{\Gamma (1+n\alpha )}t^{n\alpha }\;\left(t\to 0\right),$$

and

(111) $$L\left\{\langle |x\left(t\right){|}^{n}\rangle \right\}≃\frac{n!{\left(v\sqrt{\tau }\right)}^{n}}{s^{1+n\alpha /2}}\;\left(s\to 0\right)\;\Rightarrow {\;\langle |x\left(t\right)|}^{n}\rangle =\frac{n!{\left(v\sqrt{\tau }\right)}^{n}}{\Gamma (1+n\alpha /2)}t^{n\alpha /2}\;\left(t\to \infty \right).$$

#### 6.3.2. Two Dimensions

We now have

$$L\left\{\langle |r\left(t\right){|}^{n}\rangle \right\}=\int_{R^{2}}{\left|r\right|}^{n}\hat{p}\left(r,s\right)d^{2}r=\int_{0}^{\infty }\int_{0}^{2\pi }{\left|r\right|}^{n}\hat{p}\left(r,s\right)rdrd\varphi ,$$

that is,

$$L\left\{\langle r^{n}\left(t\right)\rangle \right\}=2\pi \int_{0}^{\infty }r^{n+1}\hat{p}\left(r,s\right)dr.$$

Substituting for Equation (90) and a simple change of variables yields

$$L\left\{\langle r^{n}\left(t\right)\rangle \right\}=\frac{v^{n}}{s{\left(s^{2\alpha }+s^{\alpha }/\tau \right)}^{n/2}}\int_{0}^{\infty }z^{n+1}K_{0}\left(z\right)dz,$$

but [70]

$$\int_{0}^{\infty }z^{n+1}K_{0}\left(z\right)dz=2^{n}\Gamma^{2}\left(1+n/2\right),$$

so that

(112) $$L\left\{\langle r^{n}\left(t\right)\rangle \right\}=\frac{2^{n}\Gamma^{2}\left(1+n/2\right)v^{n}}{s{\left(s^{2\alpha }+s^{\alpha }/\tau \right)}^{n/2}},\;\left(n=1,2,\cdots \right).$$

Observe that this two-dimensional expression is equal to the one-dimensional moment (107) except for a mere numerical factor and, therefore, all two-dimensional expressions can be recovered from the one dimensional ones under the replacement n!→$2^{n}\Gamma^{2}\left(1+n/2\right)$. Thus, in particular, we see from Equations (110) and (111) that

(113) $$\langle r^{n}\left(t\right)\rangle ∼t^{n\alpha }\;\left(t\to 0\right)\;\;\mathrm{and}\;\;\langle r^{n}\left(t\right)\rangle ∼t^{n\alpha /2}\;\left(t\to \infty \right).$$

#### 6.3.3. Three Dimensions

In the three dimensional case we have

$$L\left\{\langle |r\left(t\right){|}^{n}\rangle \right\}=\int_{R^{3}}{\left|r\right|}^{n}\hat{p}\left(r,s\right)d^{3}r=\int_{0}^{\infty }\int_{0}^{\pi }\int_{0}^{2\pi }{\left|r\right|}^{n}\hat{p}\left(r,s\right)r^{2}\sin\theta drd\theta d\varphi ,$$

that is,

$$L\left\{\langle r^{n}\left(t\right)\rangle \right\}=4\pi \int_{0}^{\infty }r^{n+2}\hat{p}\left(r,s\right)dr.$$

Substituting for Equation (91) and elementary integration yields

(114) $$L\left\{\langle r^{n}\left(t\right)\rangle \right\}=\frac{\left(n+1\right)!v^{n}}{s{\left(s^{2\alpha }+s^{\alpha }/\tau \right)}^{n/2}},\;\left(n=1,2,\cdots \right),$$

which has the same structure as the one and two dimensional cases (cf. Equations (107) and (112)) and, as a consequence, the asymptotic expressions for moments will also be given by Equation (113).

## 7. Concluding Remarks

We have reviewed the main aspects of telegraphic transport processes which account for “diffusion with finite velocity” [8] and whose master equation is the telegrapher’s equation instead of the diffusion equation. The main part of this report is a comprehensive account of our previous works [30,31,32], on the derivation, out of random walks models, of the telegrapher’s equation (ordinary as well as fractional) in one, two and three dimensions.

We have mostly focussed on two and three dimensions because, for one hand, early attempts to derive higher dimensional TE’s from random walk models had been fruitless and, on the other hand, higher dimensional models are usually more relevant for transport problems than any one-dimensional model. We thus present models that are two and three dimensional generalizations of the persistent random walk on the line. The models are based on multistate random walks with a continuous number of states representing the different directions the particle can take. We set the general integral equations for the probability density function of the particle evolution on the plane or in the space. When at every point all possible directions are independent and do not depend on the orientation and position (isotropy and homogeneity), the general equations can be exactly solved in Fourier-Laplace space. The isotropic and homogeneous models are suitable for addressing the transport of particles experiencing elastic collisions with fixed centers randomly distributed such as photons moving in turbid media [2].

These continuous models constitute a microscopic description for transport in which we statistically count the (elastic) collisions of the particles. If we zoom out this microscopic description by implementing the fluid-limit approximation of large times and distances—and, thus, going to a mesoscopic description of the process—we end up with the telegrapher’s equation as the master equation of the transport processes.

We have also generalized the telegrapher’s equation to account for anomalous transport in several dimensions. To this end the isotropic and homogeneous random walk has been extended to allow for fractional behavior both in time and space variables. The dual character of the ordinary TE between wave and diffusion behaviors is also manifest in the space-time fractional TE where at small times this equation reduces to the fractional diffusion-wave equation while at long times it does to the anomalous diffusion equation.

The two different dynamics governing the fractional transport—one of them, ruling transport at small times, is given by fractional wave behavior, while at large time the dynamics is dictated by fractional diffusion behavior—are even more apparent for the time-fractional transport when only the time variable is fractional. In this case all moments of the distance to the initial position (the effective distance travelled by the particle) exist and have an analytical expression in terms of their Laplace transforms. For small and large times these moments are approximated by

$$\langle r^{n}\left(t\right)\rangle ∼t^{n\alpha }\;\left(t\to 0\right),\;\;\langle r^{n}\left(t\right)\rangle ∼t^{n\alpha /2}\;\left(t\to \infty \right),$$

(n=1,2,⋯). When 0<α<1/2 there is a transition from two different subdiffusive regimes, while if 1/2<α<1 the transition is from superdiffusion at small times to subdiffusion at large times. This fact generalizes the passage from ballistic motion to normal diffusion shown by the ordinary telegrapher’s equation.

The exact solution for the characteristic function $\tilde{p}\left(\omega ,t\right)$ of the fractional transport has also been obtained regardless the dimensionality of the process, and approximate expressions for wave and diffusion regimes are attained as well. These variety of expressions have been explored by Mainardi and collaborators [52,53,67,72] on solutions for fractional diffusion and fractional wave-diffusion equations (see also Mainardi’s recent and useful survey appeared in this special issue [73]). Additionally, Orsingher and collaborators [74,75,76,77,78] have proposed several kinds of solutions to the fractional TE and explored their properties.

For the time-fractional transport we have been able to go one step further and obtain the exact form of the Laplace transform $\hat{p}\left(r,s\right)$ in one, two and three dimensions. From these expressions it is possible to get analytical forms of the PDF p(r,t) valid for sufficiently long times which, in one and three dimensions are written in terms of Wright functions (cf. Equations (92) and (103)), while in two dimensions by a logarithmic function (cf. Equation (99)). From these expressions we have obtained, as t→∞, the asymptotic power laws

$$p\left(x,t\right)∼t^{-\alpha /2},\;\left(\mathrm{one}\;\mathrm{dimension}\right);\;\;p\left(x,t\right)∼t^{-\alpha },\;\left(\mathrm{three}\;\mathrm{dimensions}\right);$$

and the logarithmic power law

$$p\left(x,t\right)∼t^{-\alpha }\ln t,\;\left(\mathrm{two}\;\mathrm{dimensions}\right).$$

Let us finish by recalling that a substantial part of this paper is a review of previous works but a significant part is, to my knowledge, new. This is the case of the higher dimensional extension of the characteristic function for the space-time fractional TE (cf. Section 5.4), as well as the whole Section 6 on higher dimensional time-fractional telegraphic processes.
